# Adaptation of land management in the Mediterranean under scenarios of irrigation water use and availability

**DOI:** 10.1007/s11027-017-9761-0

**Published:** 2017-09-10

**Authors:** Žiga Malek, Peter H. Verburg

**Affiliations:** 1 Institute for Environmental Studies (IVM), VU University Amsterdam, De Boelelaan 1085, 1081HV Amsterdam, The Netherlands; 2 Swiss Federal Institute for Forest Snow and Landscape Research, WSL Zürcherstrasse 111, 8903 Birmensdorf, Switzerland

**Keywords:** Agricultural intensification, Global change, Irrigation efficiency, Land management, Land systems, Multifunctionality, Water resources

## Abstract

Meeting the growing demand for food in the future will require adaptation of water and land management to future conditions. We studied the extent of different adaptation options to future global change in the Mediterranean region, under scenarios of water use and availability. We focused on the most significant adaptation options for semiarid regions: implementing irrigation, changes to cropland intensity, and diversification of cropland activities. We used Conversion of Land Use on Mondial Scale (CLUMondo), a global land system model, to simulate future change to land use and land cover, and land management. To take into account future global change, we followed global outlooks for future population and climate change, and crop and livestock demand. The results indicate that the level of irrigation efficiency improvement is an important determinant of potential changes in the intensity of rain-fed land systems. No or low irrigation efficiency improvements lead to a reduction in irrigated areas, accompanied with intensification and expansion of rain-fed cropping systems. When reducing water withdrawal, total crop production in intensive rain-fed systems would need to increase significantly: by 130% without improving the irrigation efficiency in irrigated systems and by 53% under conditions of the highest possible efficiency improvement. In all scenarios, traditional Mediterranean multifunctional land systems continue to play a significant role in food production, especially in hosting livestock. Our results indicate that significant improvements to irrigation efficiency with simultaneous increase in cropland productivity are needed to satisfy future demands for food in the region. The approach can be transferred to other similar regions with strong resource limitations in terms of land and water.

**Electronic supplementary material:**

The online version of this article (10.1007/s11027-017-9761-0) contains supplementary material, which is available to authorized users.

## Introduction

Irrigated cropland has a significant share in the global crop production and allows human presence and a stable crop production in areas otherwise inhabitable (Evans and Sadler 2008). In the light of future climate and population change, more existing cropland will need to be equipped with irrigation in order to maintain higher yields or crop production in general (UNESCO 2006). This is expected to have substantial effects on freshwater resources. Already today, irrigation is the largest consumer of freshwater resources and has led to unsustainable water withdrawals in several world regions (Wisser et al. 2008). In the coming decades, more than a half of the global population is expected to live under conditions of water scarcity (Qadir et al. 2007). Improving the irrigation efficiency has been identified as a major strategy to adapt to future climate and socioeconomic change globally and in major arid regions (Smit and Skinner 2002; Fader et al. 2016).

More efficient irrigation is only one aspect of adaptation to global change (Elliott et al. 2014). Besides reducing water losses, other improvements in land management are necessary to ensure food security and mitigate environmental impacts of agriculture (de Fraiture and Wichelns 2010; Neumann et al. 2010; Mueller et al. 2012). Although increased productivity would result in significantly higher crop production, it remains unclear how changes to land management can contribute to this increase (Licker et al. 2010). Integrated approaches on adaptation responses in land management may help target investments and estimate the potential contributions of alternative strategies from a system-wide perspective rather than only at the farm level (Falkenmark et al. 2014). Many studies have addressed how future global change might result in changes to land use and land cover, and land management (Letourneau et al. 2012; van Asselen and Verburg 2013; Eitelberg et al. 2016). While most land change studies did account for climate change, they did not treat water as a limiting resource and consider potential improvements to irrigation efficiency. Studies evaluating improvements to irrigation efficiency did not investigate other simultaneous adaptation in land management (Rost et al. 2009; Elliott et al. 2014; Fader et al. 2016). In this study, we evaluate water and land management strategies to adapt to future global change. We focus on the Mediterranean, a region under water stress with high requirements for irrigation (Wriedt et al. 2009; Hayashi et al. 2013).

The Mediterranean is a densely populated semiarid region, with harsh water and land resources constraints (Giannakopoulos et al. 2009; Fader et al. 2016). While the Mediterranean north is a significant exporter of agricultural commodities (Daccache et al. 2014), the Middle East and North African part depends heavily on food imports (Wright and Cafiero 2011). Fluctuations in food supply and prices are threatening food security and increasing the social and political vulnerability in the region (Sowers et al. 2010). Projected climate changes suggest an aridity increase and a decrease in freshwater resources, impacting future crop production (Vörösmarty et al. 2010; Chenoweth et al. 2011; Guiot and Cramer 2016). At the same time, the irrigation water withdrawal in the region is expected to increase (Daccache et al. 2014; Salmoral et al. 2017). Reducing water stress and depletion of freshwater resources, while increasing food production, have therefore been recognized as main future challenges of the region (UNEP/MAP-Plan Bleu 2010).

In this paper, we present a novel land system modeling application to evaluate adaptation options using a regional, spatial perspective rather than focusing on the field or farm level. We combine global scenarios of socioeconomic and climate change with regional irrigation characteristics and land use configuration. Our objective is to evaluate irrigation and land management strategies to adapt to future changes to climate and water resources in the Mediterranean region. Particularly, we analyze how reducing water loss and irrigation water withdrawals affects future expansion of irrigated areas, at the same time leading to changes in land management. This way, we study the extent of changes in cropland intensity, cropland expansion, and diversification of agricultural activities necessary to satisfy future food demand in the Mediterranean region under different levels of achieved irrigation efficiency.

## Methodology

### The Mediterranean region

**Fig. 1:**
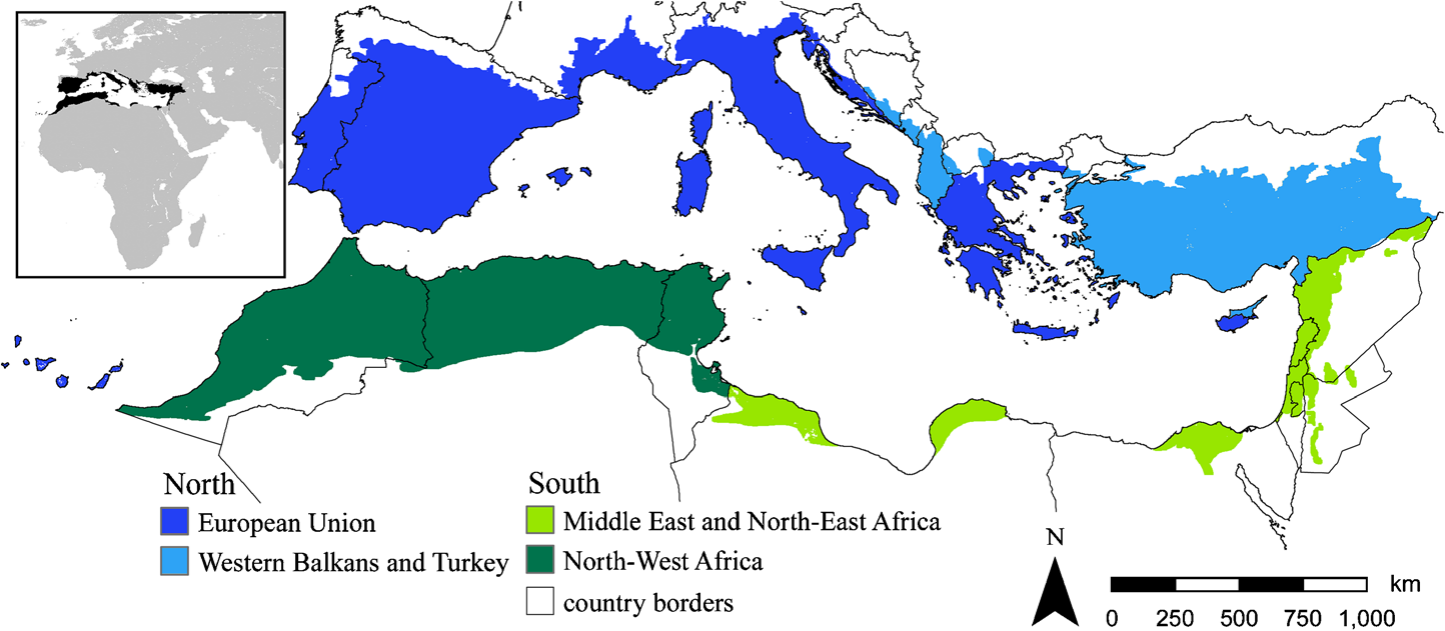
The study area with four thematic subregions

The Mediterranean region spans over southern Europe, Northern Africa, and the Middle East. We focus on the Mediterranean ecoregion (Fig. 1), which describes the approximate original extent of representative Mediterranean natural communities (Olson et al. 2001). It covers 2.3 million km^2^ in 27 countries with around 420 million inhabitants. We divided the region thematically to the northern and the southern part with further subdivisions into two subregions each (Fig. 1). This way, we captured regionally specific characteristics of land use and land cover (S1), and their irrigation water withdrawal demands on a higher detail. There are considerable differences in freshwater resources, and type of irrigation within the region, particularly between the northern and southern part of the Mediterranean (Table 1). Overall, irrigation amounts to 69% of total water withdrawal in the region, making it the largest water consumer in the region (FAO 2016).

**Table 1:** Water resources and irrigation system characteristics. Data from UNEP/MAP-Plan Bleu (2010), EUROSTAT (2013) and FAO (2016)

Region	Water resources and irrigation withdrawal				Share of irrigation systems in %		
	Irrigation water as % of total water withdrawal	Irrigation water withdrawal (km^3^/year)	Pressure on freshwater resources (%)	Reported overall irrigation efficiency (%)	Surface	Sprinkler	Drip
North							
Western Balkans and Turkey	78.9	30.1	11.8	53.7	88.0	9.2	2.8
European Union	39.3	48.6	10.2	65.4	42.1	29.7	24.4
South							
Middle East	93.8	59.3	94.4	62.0	82.9	8.0	9.1
NW Africa	78.2	16.7	30.2	66.2	81.7	11.7	6.6

### CLUMondo model

CLUMondo (Conversion of Land Use on Mondial scale) is a spatial land system change model, where future changes to the landscape are driven by multiple demands (S2), such as crops, livestock, and built-up areas (van Asselen and Verburg 2013). The model goes beyond simulating land cover changes only, as land systems combine information on land management, fertilizer input, yield gap, and livestock numbers (Souty et al. 2012; van Asselen and Verburg 2013). The explicit representation of land management allows accounting for change in the intensity of production systems, for example, by improving yields or increasing livestock density (van Asselen and Verburg 2013). This is necessary, as socioeconomic changes often do not impact land cover directly, but result in changes in management intensity.

CLUMondo allocates changes to land systems based on local spatial preference, area restrictions, and competition between land systems (van Asselen and Verburg 2013). We calculated spatial preference of land systems by investigating the relationships between their spatial occurrence (S1) and explanatory biophysical and socioeconomic variables (S3) using logistic regression (S4). Area restrictions are spatial limitations, such as protected areas. We used the current network of protected areas (IUCN 2015) to restrict intensification and cropland and urban expansion in protected areas. The model satisfies the demand by promoting the most competitive and productive land systems by iteratively calculating optimal land system allocation provided the constraints and location preferences.

CLUMondo can also consider the demands of systems for specific resources, such a freshwater. In this study, water withdrawal was constrained by applying a threshold on maximum water withdrawal. This innovative concept has a significant effect on allocating future land system change, as the model cannot only promote the most competitive systems in terms of output without taking into account actual available water resources. The model has to allocate land systems with lower output that have low or no irrigation demands. The model is described in more detail by van Asselen and Verburg (2013) and is available at http://www.environmentalgeography.nl/site/data-models/data/clumondo-model/.

We used the Mediterranean land systems map for the year 2010 (S1, Malek and Verburg 2017) as a starting point to simulate future land system change until 2050. Land systems were defined as combinations of land cover (cropland extent, tree cover density, extent of bare and built-up areas), management (crop type, irrigation, intensity), and livestock presence on a 2 × 2 km resolution. The average output of annual and permanent crops per land system unit was based on agricultural production statistics for 2010 (S2), and the share of crops produced in systems with low and high intensity and in systems under irrigation (EUROSTAT 2013, 2016; You et al. 2014). We changed the output of land systems annually to account for cropland productivity increases. The average yield for each land system was based on yield gap data (Foley et al. 2011) and served as our reference. The irrigation demand of the different land systems was based on the average extent of areas equipped with irrigation of each land system (Siebert et al. 2005, 2013), and national and subnational irrigation water withdrawal statistics (EUROSTAT 2013, 2016; FAO 2016). This way, we obtained mean values of irrigation water withdrawal per cell of irrigated land system (S2).

### Future population, climate, and food demand

Future population change (Table 2) followed the Shared Socioeconomic Pathways (SSP) scenarios SSP2 population projections, where the existing 2010 population map was updated annually until 2050 (CIESIN 2015; Jiang and O’Neill 2017). Growth rates for urban population were used for areas with high population density (> 250 inhabitants/km^2^, S5), for other areas, we used growth rates for rural population (Jiang and O’Neill 2017).

**Table 2:** Changes to population, climate, and food production demands in the Mediterranean for the scenario up to 2050 based on the SSP2 Marker scenario

	North	South
Population and built-up areas	+ 19.5%	+ 47.4%
Climate change	RCP4.5 (S2)	RCP4.5 (S2)
Annual and permanent crops	+ 18.1%	+ 40.2%
Livestock	+ 25.8%	+ 23.8%

To consider climate change, we used projections from downscaled global climate models from the CMIP5 (Coupled Model Intercomparison Project), forced by the Representative Concentration Pathway 4.5 (RCP4.5) greenhouse gas radiative forcing (Hijmans et al. 2005; Taylor et al. 2012). We calculated the mean of 19 CMIP5 simulations (S6) for temperature and precipitation. We prepared annual temperature and precipitation maps, using 2050 values (mean of 2041–2060). These maps were used to derive potential evapotranspiration (PET) and aridity index (AI) maps (Trabucco et al. 2008; Zomer et al. 2008) (S7). AI was used as a limiting factor for particular land system change processes. For example, forest expansion on abandoned cropland was only possible in areas with AI > 0.65 (Zomer et al. 2008). Mediterranean forests consist mostly of oak (*Quercus sp.*) and pine (*Pinus sp*.) forests, and although they are used to the semiarid Mediterranean climate, they mostly occur in hilly and mountainous areas with sufficient rainfall (Malek and Verburg 2017).

Future demands for annual and permanent crops, livestock, and built-up area (Table 2) followed the SSP2 Marker scenario (Fricko et al. 2017; Riahi et al. 2017). These projections result from global economic models that roughly account for regional production capacity, consumption, and trade. It should be noted that these demands do not aim to fully fulfill food requirements of the region: trade and imports are an important component and integrated in the demands used in our study. In this study, annual crops contain major cereals like wheat (*Triticum aestivum*), barley (*Hordeum vulgare*), maize (*Zea mays*), and rice (*Oryza sativa*), together with vegetables including tomatoes (*Solanum lycopersicum*) and potatoes (*Solanum tuberosum*). Permanent crops contain fruit such as apples (*Malus sp*.), pears (*Pyrus sp*.), peaches (*Prunus persica*), grapes (*Vitis vinifera*), citrus (*Citrus sp*.), olives (*Olea europaea*), and dates (*Phoenix dactylifera*). Livestock numbers consist only of ruminant species: cattle (*Bos taurus*), goats (*Capra aegagrus*), and sheep (*Ovis aries*). The production of annual and permanent crops is based on food production projections in the SSP2 Marker scenario and livestock production on projected livestock numbers (IIASA 2016; Riahi et al. 2017). The demand for built-up areas is linked to population change (Kc and Lutz 2017; IIASA 2016). Technical details of translating the demands from global projections to the CLUMondo land system model are described in S8.

### Irrigation efficiency and water withdrawal scenarios

Defining the reference situation of irrigation efficiency remains a challenge due to different definitions (Jensen 2007; Evans and Sadler 2008; Jägermeyr et al. 2015). When describing the efficiency of irrigation regimes, the term field application efficiency is normally used. It describes the share of water that reaches the field and is relatively high in the Mediterranean region (Lankford 2006; Evans and Sadler 2008). Other losses are associated with water transport from the source to the field and are defined as conveyance efficiency (Evans and Sadler 2008; Jägermeyr et al. 2015). Most water losses in the Mediterranean region are related to the conveyance efficiency, i.e., due to inefficient transport infrastructure (Sowers et al. 2010).

In this study, we focused on overall irrigation efficiency. It describes the share of water used efficiently and the share that is lost from the total water extracted from the source of supply on a larger, national scale (UNEP/MAP-Plan Bleu 2010; FAO 2016). The overall efficiency of a scenario was calculated as a product of field application efficiency and conveyance efficiency, using the efficiencies proposed by Fader et al. (2016). Reported overall irrigation efficiencies were used as our reference scenario (FAO 2016), to consider existing cumulative regional water loss (Table 1).

We added two scenarios with higher overall irrigation efficiencies, based on literature and reported efficiencies (UNEP/MAP-Plan Bleu 2010; Jägermeyr et al. 2015; FAO 2016). Governments in the region have made significant effort in the past years to replace surface irrigation systems with more efficient sprinkler or drip irrigation systems, which influenced our scenario assumptions (Wriedt et al. 2009; Daccache et al. 2014). In the “sprinkler” and “drip” scenario, overall irrigation efficiency is assumed to reach the level reported for the respective system (Table 3). We ran an additional “deficit irrigation” scenario (Table 3), where crops are allowed to experience mild water stress with marginal decreases in yield and quality (Costa et al. 2007; Wriedt et al. 2009). Regulated deficit irrigation has been identified as a complementary approach to increase water savings (Geerts and Raes 2009). It represents a low-cost scenario, where no significant improvements to irrigation infrastructure are needed.

**Table 3:** Overview of irrigation efficiency scenarios

Scenario	Description
Reference	No improvements to irrigation efficiency and no decrease in water losses
Sprinkler	Improvements to overall irrigation efficiency to decrease regional water loss to 28.7% of total water withdrawal. Based on the efficiency of sprinkler irrigation (Fader et al. 2016)
Drip	Improvements to overall irrigation efficiency to decrease regional water loss to 14.5% of total water withdrawal. Based on the efficiency of drip irrigation (Fader et al. 2016)
Deficit irrigation	No improvements to irrigation efficiency and no decrease in water loss. 27.4% reduction in water used on irrigated cropland, together with a 4% decrease in total output of irrigated cropland (Wriedt et al. 2009)

To simulate the scenarios, we changed the water demand of irrigated land systems for the two irrigation efficiency scenarios (S2). For the deficit irrigation scenario, we decreased the water demand for the irrigated land systems, while reducing their total output for crops (Table 3). All scenarios had the same improvement in crop efficiency, resulting in reduced yield gaps. We increased the yield of rain-fed intensive cropland systems to 75% of the potential yield and to 90% for irrigated cropland. The values are based on plausible improvements in cultivars, mechanization, and nutrient management as proposed by Mueller et al. (2012). We ran all irrigation efficiency scenarios with two irrigation water withdrawal levels, resulting in eight scenarios (Table 3). We first simulated future land systems distribution with current irrigation withdrawal values (Table 1). Then, we reduced water withdrawal by 25% until 2050. The two irrigation withdrawal levels enabled us to compare future pathways where only irrigation efficiency is improved with the ones where water extraction levels are actually reduced.

### Adaptation options in land management

The methods used and their inherent capabilities enabled us to focus on large scale adaptation in land management: implementing irrigation, changes to cropland intensity, and changes to the number of agricultural activities within the same landscape (Table 4). Most significant adaptation responses in land management in the region are related to improvements in the efficiency of water use and cropland intensification (Sowers et al. 2010). Due to the significance for cultural heritage and biodiversity embedded in Mediterranean mosaic land systems (Médail and Quézel 1999; Tieskens et al. 2017), we specifically look at adaptation related to these systems. Mediterranean mosaics are multifunctional, agro-silvo-pastoral systems that have developed through centuries adapting to harsh environmental conditions (Médail and Quézel 1999). One example are the *dehesa*s/*montado*s of Spain and Portugal, open woodlands mostly consisting of oaks (*Quercus ilex* and *Quercus suber*). *Dehesas/montados* are primarily used for grazing of cattle (*Bos taurus*), sheep (*Ovis aries*), goats (*Capra aegagrus*), and also pigs (*Sus scrofa domesticus*). They provide valuable non-timber forest products such as cork, firewood, and acorns. In the understory layer, cereals such as wheat (*Triticum aestivum*), oats (*Avena sativa*). or barley (*Hordeum vulgare*) are often cultivated. Grazing, cereal cultivation, and forestry therefore occur at the same time in these landscapes (Joffre et al. 1999).

**Table 4:** Studied adaptation options

Adaptation option	Description	Land system conversions
Implementing irrigation	Equipping cropland with irrigation	Rain-fed to irrigated cropland
Changes to cropland intensity	Increase: increase in the intensity of rain-fed cropland	Extensive cropland to intensive rain-fed cropland
	Decrease: decrease in the intensity of cropland activities	Intensive rain-fed cropland to extensive cropland
	Expansion: introducing cropland activities	Non-cropland to cropland land system
	Abandonment: abandonment of cropland activities	All cropland types to non-cropland land system
Diversification	Introducing new activities on cropland or woodlands, e.g. livestock grazing	Mono-functional systems to multifunctional mosaic systems
Changing functionality	Changing activities in multifunctional landscapes	Conversions within multifunctional mosaic systems

## Results

**Fig. 2:**
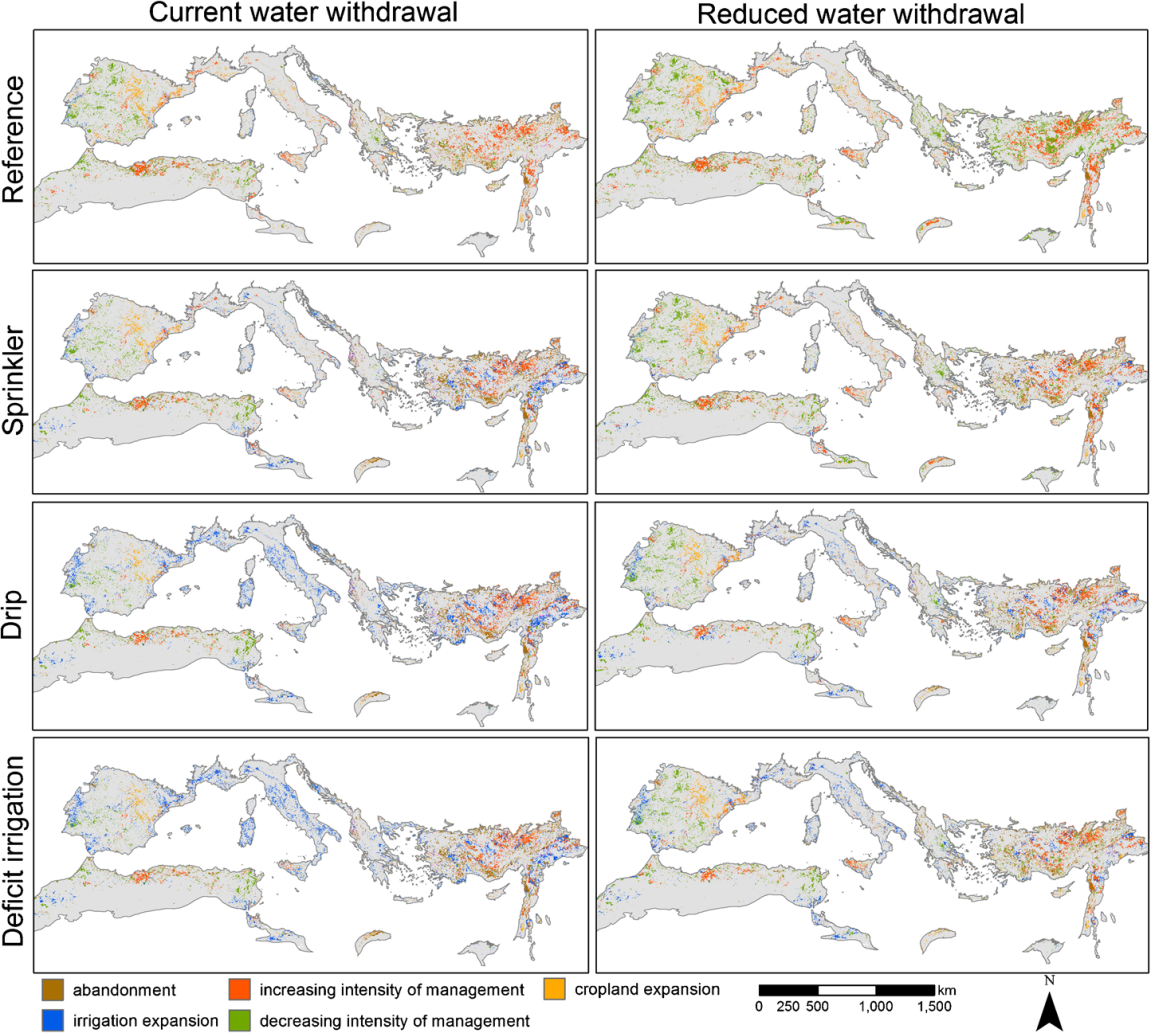
Changes in cropland cover, irrigation, and intensity under scenarios of irrigation efficiency and water withdrawal

**Fig. 3:**
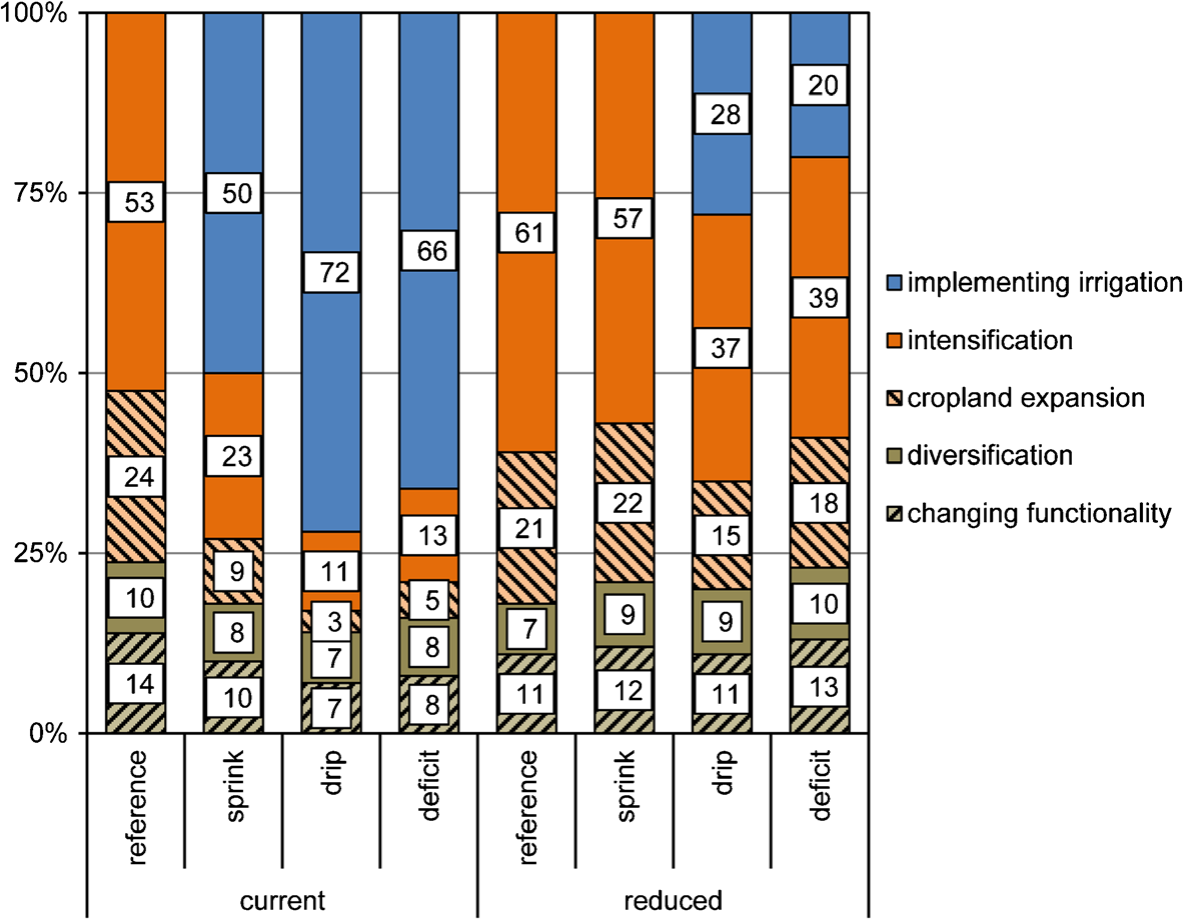
Adaptation options contributing to future increases in food demand (%). No values occur when adaptation does not contribute to additional food demand

**Fig. 4:**
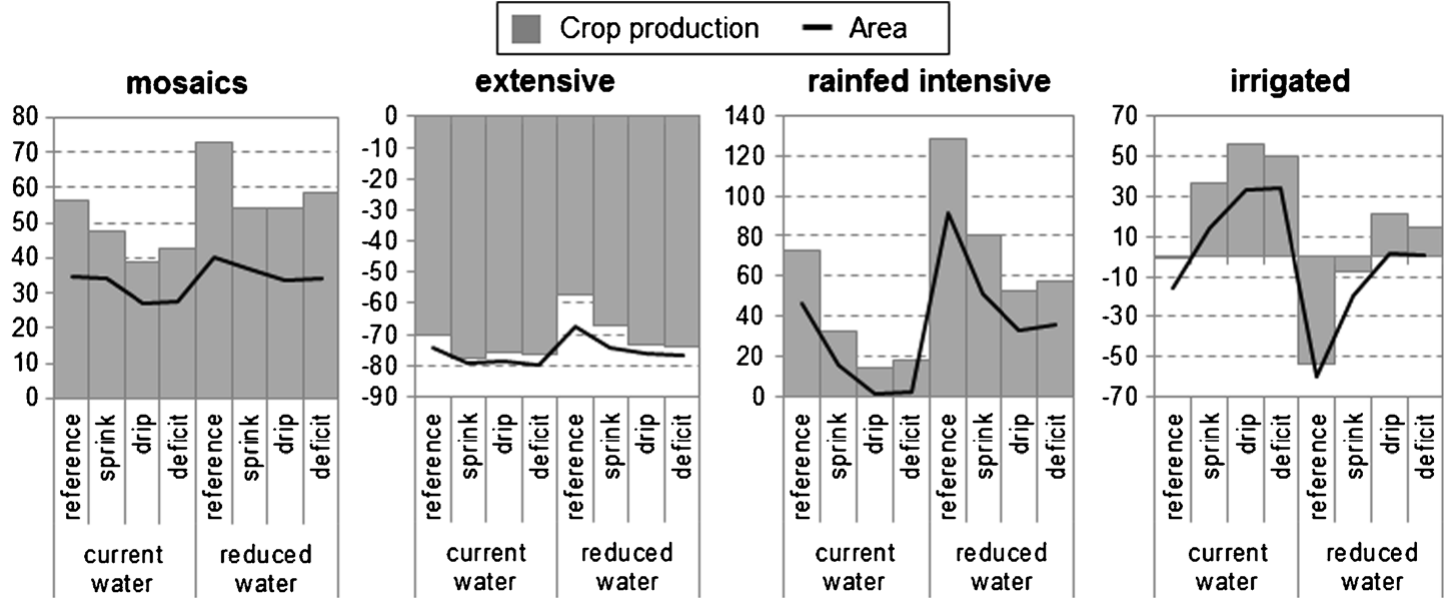
Change to areas and crop production (%) under scenarios of irrigation efficiency and water withdrawal

**Fig. 5:**
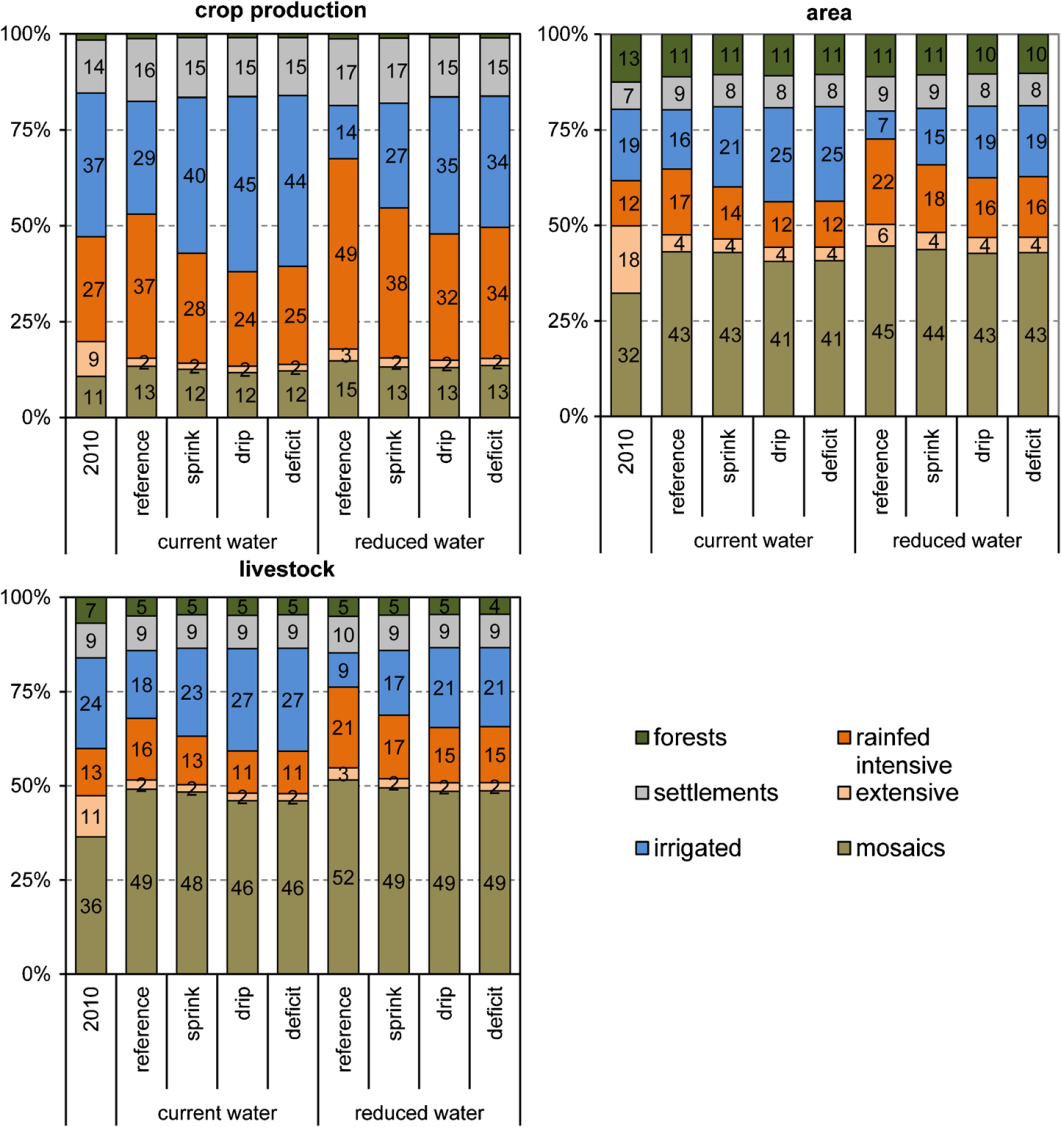
Share of land systems in crop production, area, and livestock under scenarios of irrigation efficiency and water withdrawal

Spatial distributions of future land system change under different scenarios are summarized in Fig. 2. The extent of each adaptation option contributing to future food demand is presented in Fig. 3. Figures 4 and 5 describe the changes to major land system groups in terms of crop production, area covered, and livestock. All scenario results are presented in the Supporting information (S9 and S10) and are available in GIS format on www.environmentalgeography.nl.

### Implementing irrigation

Generally, there is significantly less expansion of irrigated cropland in scenarios with stronger restrictions on water withdrawal (Figs. 2 and 4). In the reference scenario, irrigated cropland cannot contribute to future increases in food demand (Fig. 3), and even results in a decrease in total irrigated area and crop production Figs. 4 and 5). Similar trends can be observed for the sprinkler scenario for both levels of water withdrawal. While future increases in area and crop production are possible with the current level of water withdrawal, the sprinkler scenario also results in a decreased extent of irrigated cropland in the case of reduced water withdrawal (Figs. 3 and 4).

Both high irrigation efficiency scenarios indicate that substantial improvements to irrigation systems are necessary if the Mediterranean wants to continue satisfying most of its future food demand with irrigated crops (Fig. 3). The large potential of drip and deficit irrigation is illustrated by the overall increase in crop production in irrigated systems under both water withdrawal levels for these scenarios (Fig. 4). This is mostly on the account of the assumed improved productivity of irrigated cropland. In case of reduced water withdrawal, the share of irrigated cropland in future crop production needs to be considerably lower, despite improvements in irrigation efficiency (Fig. 3).

### Changes to cropland intensity

Increases in the intensity of rain-fed cropland will be necessary, particularly in the case of reduced water withdrawal (Figs. 3 and 4). Substantial cropland intensification is projected in areas currently defined by a large share of low intensity cropland, such as Algeria, Syria, and Turkey (Fig. 2). In the Northern Mediterranean and Tunisia, a decrease in cropland intensity is occurring simultaneously with cropland expansion (Fig. 2). Cropland abandonment (S10) and shift to other areas in these parts of the region can be explained by climate change and marginality of some of the current cropland. All scenarios project a drastic reduction of extensive cropland in favor to more intensive systems and abandonment, with actual cropland expansion playing a minor role (Fig. 3, S10).

Scenarios with reduced water withdrawals present futures where considerably more crops need to be produced on intensive rain-fed cropland (Fig. 3). How much rain-fed intensive cropland will contribute to future food production through intensification and expansion depends on the level of water used efficiently, and ranges between 14 and 82% (Fig. 3). Increased productivity of rain-fed cropland will be particularly necessary in case of no or low improvements to the irrigation efficiency. The results indicate that, for instance, the spatial extent of intensive rain-fed cropland will need to almost double (+ 91%) in case of no irrigation efficiency improvements and reduced water withdrawals (Fig. 4).

### Diversification of agricultural activities and changes to multifunctionality

While across the world multifunctional agricultural systems are under pressure, our results project an expansion of mosaic land systems in all scenarios. The area of these multifunctional systems increases by 27–40% and total crop production in these systems by 39–73% (Fig. 4). Under current water withdrawal, mosaic systems expand mostly on extensive cropland (Table 5). Reducing water withdrawal results in adaptation through diversifying agricultural activities on irrigated areas, as intensive rain-fed cropland is often not possible on such locations. The share of irrigated cropland converted to mosaics is most striking in case of no improvements to irrigation efficiency (Table 5). Despite the overall net increase of mosaic systems, around a third of them change in all scenarios. Interestingly, more mosaic systems are persistent in both low irrigation efficiency scenarios (reference and sprinkler) under current water extraction levels. Most of mosaics change to another mosaic system, and only 4.2 to 6.6% are actually lost by being converted to other land systems (Table 5).

**Table 5:** Share of land systems with allocated diversification (%) and changes to the multifunctionality (MF) in mosaic systems (% of 2010)

	Reference		Sprinkler		Drip		Deficit	
	Current	Reduced	Current	Reduced	Current	Reduced	Current	Reduced
Diversification of cropland systems								
Extensive rain-fed cropland	52.7	41.3	62.3	53.1	64.0	54.3	64.2	55.8
Intensive rain-fed cropland	17.3	10.0	19.2	15.0	17.6	14.3	19.4	16.1
Irrigated cropland	14.3	40.4	6.0	20.0	3.1	15.3	2.8	15.4
Changes to mosaic systems								
Persistent mosaics	68.6	65.0	66.5	66.3	64.2	65.9	63.9	65.8
Reduced MF	7.7	6.0	7.4	6.5	8.6	7.1	8.2	5.7
Same MF level, different system	15.4	15.8	17.8	17.0	17.7	18.0	17.5	17.4
Increased MF	8.3	13.2	8.3	10.2	9.5	9.0	10.4	11.2
Converted to other system	4.2	6.6	4.2	5.1	4.7	4.5	5.2	5.6

Although mosaic systems satisfy up to a fourth of additional food demand in the future (Fig. 3), their share in total crop production remains low (Fig. 5). These systems are low intensity systems with a lower crop output compared to single function systems. Additionally, we assumed a stable agricultural production within these traditional systems, as they are unlikely to benefit from technical improvements opposed to intensive agricultural systems. The role of mosaic systems is more significant when looking at their livestock production. Already at the baseline, they host a third of all bovines, goats, and sheep in the Mediterranean, reaching almost a half of total livestock production in the future (Fig. 5). The model mostly converted extensive cropland to agro-silvo-forestry systems with higher livestock densities (Table 5). Conversions to other mosaic systems (e.g., from cropland-woodland to cropland-wooded rangeland) projected an intensification of livestock grazing in mosaics (Table 5). This can lead to adverse impacts on the other functions and values of these traditional landscapes.

## Discussion

### Future adaptation in land management

The limited amount of freshwater constraints the extent of irrigated cropland in the Mediterranean and stimulates non-irrigation adaptation options to future global change. Other land systems, characterized by low intensity but multifunctional in nature, will keep on having a significant role in future food production of the region. Generally, reducing water losses leads to further expansion of irrigated systems and increases of crops produced in irrigated areas. Existing irrigated cropland was projected to decrease in case of no or low irrigation efficiency improvements (reference and sprinkler). Although a reduction in irrigated areas seems unrealistic, it is occurring on a local scale. Crops with significant water needs such as citruses are being substituted with crops with lower or no irrigation demands (Iglesias et al. 2011).

Our scenarios project a significant increase in intensity of rain-fed cropland, particularly in the southern Mediterranean and Turkey, which is also supported by global studies (Mueller et al. 2012). Climatic conditions, variability in precipitation, and extreme weather events may limit such intensification in practice. The large extent of cropland intensification projected by the low irrigation efficiency scenarios is unlikely and more food imports might be needed (Rudel et al. 2009).

Maintaining multifunctional mosaic land systems, as well as introducing additional activities to low intensity croplands can be considered as an additional adaptation strategy to scarce water resources. These systems are mostly satisfying the increased demand for livestock and partially for crops. Such traditional systems may play a significant role in mitigating and preventing other negative consequences of intensive agriculture, such as soil erosion (Almagro et al. 2016) or biodiversity loss (Fagúndez et al. 2016), and are particularly adapted to the hilly areas with high variability in environmental conditions. Increasing and maintaining the extent of such land systems will be difficult. Local studies demonstrate that these areas are subject to abandonment and loss due to intensification (Bajocco et al. 2012; Schaich et al. 2015). Moreover, future climate change might reduce the crop and livestock productivity of these systems (Latorre et al. 2001; Freier et al. 2014).

### Discussion of the methodology

We presented a novel approach, where land system modeling is used to investigate future adaptation in land management under different irrigation efficiency and water withdrawal scenarios. The scenarios were based on plausible improvements to the overall irrigation efficiency (Fader et al. 2016) and deficit irrigation (Wriedt et al. 2009). We applied rather modest water savings and impacts on yields, as higher water savings are possible when changing crops or improving cultivars (Costa et al. 2007). Much higher losses to yields could be possible due to future climate change (Moriondo et al. 2011). Furthermore, an increase in irrigation water withdrawal in the region might be possible, as suggested by integrated global assessment models (Chaturvedi et al. 2015). The need for increased water withdrawal might also be significantly higher due to increased crop water demands (Giannakopoulos et al. 2009). The scenarios therefore only cover part of the range of possible changes in circumstances. Irrespective of these uncertainties, the results illustrate the large land system consequences adaptation might have for the region. All scenarios also included improvements to yields and livestock densities. Assumptions on future advancement in agricultural technologies are subject to high uncertainty, particularly due to future climate change, and could have large consequences on the ability of the region to produce food (Asseng et al. 2013).

In this study, we did not consider other competing uses of water, such as municipal water, water for industry, or livestock. Undoubtedly, socioeconomic development in the Mediterranean will result in increased demands for water by the non-agricultural sector, a common trend in middle income and developing countries (Flörke et al. 2013). Tourism is a major user of water, competing for water resources with agriculture (Ortuño et al. 2015). Livestock also has significant demands for water, with livestock-related products often being related to a high water footprint (Mekonnen and Hoekstra 2012). To consider the competition between different water users, improved data is needed. For example, statistics on water withdrawal for the southern Mediterranean countries assign all agricultural water withdrawals to irrigation, without providing information on other agricultural water use (FAO 2016).

Demand for agricultural products was assumed to follow the integrated assessment model outcomes for the SSP2 scenario for all our scenarios. In reality, changing environmental conditions and constraints on water resources will impact the trade balance of the region. Increasing difficulty to produce in the region may limit exports and strongly increase imports. At the same time, global food markets will come under increasing pressure due to growing demands and there will be limitations to further increase imports to the region beyond those projected by the global models under the SSP2 conditions. Therefore, the scenarios presented should be seen as experiments of what is feasible in terms of meeting such demands under various constraints and improvements in the use of irrigation.

We present an approach that can be transferred to other regions experiencing similar resource constraints or aridity. We demonstrated how the amount of water available for irrigation significantly impacts the extent of future irrigation expansion and intensification of rain-fed cropland. Many (semi)arid region are projected to experience significant increases in food demand, and analyzing future change to land management in these regions should consider potential changes to water resources. We have also shown how future land system change is influence by the level of technological improvement, resulting in improved irrigation efficiency or cropland productivity. When developing scenarios with plausible technological improvements, we suggest focusing on regionally significant adaptation options. Finally, this study presents a relatively straightforward approach that can also be used in other regions, as it is mostly based on publicly accessible spatial data and statistics. Combining data on land use, management intensity, and irrigation, with crop production and water withdrawal values, however, demands a certain level of generalization, depending on the spatial scale.

### Limitations to adaptation

Meeting the future demands for agricultural production and the challenges of climate change requires more than efficient irrigation: also within the other land systems, changes in intensity and crop type will be needed. This study indicates that the extent of simulated adaptation in land management heavily depends on the level of water used efficiently. In practice, the potential of a region to produce crops that need irrigation is limited with the region’s ability to invest in irrigation. The Mediterranean south has a considerably higher share of irrigation with highest water losses (Table 1) (Jägermeyr et al. 2015). These systems have lower capital costs and energy demands (Sauer et al. 2010), and improving their efficiency is particularly costly.

We did not consider the depletion of freshwater resources due to unsustainable water extraction use and climate change, already occurring in the region (Hayashi et al. 2013). The 25% decrease in water withdrawals represents a reduction as an effect of policy and higher decreases in water withdrawals might actually be needed. The pressures on freshwater resources (PFR) did not deteriorate in any scenario (S11). Improving the irrigation efficiency commonly does not lead to reductions in water extraction but rather leads to increases in irrigated areas (Jensen 2007). This can be explained by the fact that if farmers reduce the water use per unit of cropland while receiving the same amount of water for irrigation, they will expand the extent of irrigated cropland to increase their production. There were substantial decreases in PFR when improving the irrigation efficiency in the Mediterranean south. Mostly, this is due to the assumed improvement to cropland productivity. Crop water productivity—the ratio between crop yield and consumed water—in this region has been identified among the lowest globally, with high potential for improvement (Zwart and Bastiaanssen 2004). Relatively high productivity increase in this region at the same time decreased the need to expand the extent of irrigated areas. However, in parts of this region, political instability constrains investments in more efficient and productive agriculture.

## Conclusions and recommendations

Our results show that integrated strategies are needed to improve the output of Mediterranean land systems, while preserving traditional landscapes and water resources to meet the future agricultural challenges. Available strategies have different impacts on land and water resources in a region, where land degradation and water stress already are a significant issue today. Only improving irrigation systems is not sufficient and should come together with strategies to improve or maintain productivity in all cropland systems. In the case of reduced water resources, the largest potential lies in improving the productivity of existing cropland in the Mediterranean region.

In order to expand irrigated cropland in areas where there is no cropland today, or it is being managed with low intensity, the irrigation efficiency and productivity of irrigated systems need to be improved, or existing irrigation systems need to be converted to more efficient ones. No or low improvements will not allow for significant expansion of irrigated cropland under current water withdrawal and could lead to a decrease in irrigated cropland in a situation with reduced water withdrawal. Moreover, low improvements to irrigation efficiency require extremely high increases in crop production in rain-fed land systems. Such intensification of rain-fed systems can in reality be unlikely, considering projected climate change and variability in precipitation. High agricultural supply assumed in global assessments may therefore be impossible without improvements in irrigation efficiency. Increasing the intensity of management of rain-fed cropland is necessary to lower irrigation water demands while at the same time increasing crop production. In order to reduce grazing pressure in other, also arid areas, we suggest preserving traditional Mediterranean multifunctional systems. Finally, limitations to water withdrawal are needed to reduce water stress in the region. We acknowledge that such efforts are an enormous challenge, as the region is characterized by large disparities in socioeconomic development.

## Electronic supplementary material

ESM 1 (DOCX 4426 kb)

## References

[CR1] Almagro M, de Vente J, Boix-Fayós C, García-Franco N, de Aguilar JM, González D, Solé-Benet A, Martínez-Mena M (2016). Sustainable land management practices as providers of several ecosystem services under rainfed Mediterranean agroecosystems. Mitig Adapt Strateg Glob Chang.

[CR2] Asseng S, Ewert F, Rosenzweig C, Jones JW, Hatfield JL, Ruane AC, Boote KJ, Thorburn PJ, Rötter RP, Cammarano D, Brisson N (2013). Uncertainty in simulating wheat yields under climate change. Nat Clim Chang.

[CR3] Bajocco S, De Angelis A, Perini L, Ferrara A, Salvati L (2012). The impact of land use/land cover changes on land degradation dynamics: a Mediterranean case study. Environ Manag.

[CR4] Chaturvedi V, Hejazi M, Edmonds J, Clarke L, Kyle P, Davies E, Wise M (2015). Climate mitigation policy implications for global irrigation water demand. Mitig Adapt Strateg Glob Chang.

[CR5] Chenoweth J, Hadjinicolaou P, Bruggeman A (2011). Impact of climate change on the water resources of the eastern Mediterranean and Middle East region: modeled 21st century changes and implications. Water Resour Res.

[CR6] CIESIN (2015) Gridded population of the world, version 4. Center for International Earth Science Information Network-CIESIN-Columbia University. NASA Socioeconomic Data and Applications Center (SEDAC)

[CR7] Costa JM, Ortuño MF, Chaves MM (2007). Deficit irrigation as a strategy to save water: physiology and potential application to horticulture. J Integr Plant Biol.

[CR8] Daccache A, Ciurana JS, Diaz JAR, Knox JW (2014). Water and energy footprint of irrigated agriculture in the Mediterranean region. Environ Res Lett.

[CR9] de Fraiture C, Wichelns D (2010). Satisfying future water demands for agriculture. Agric Water Manag.

[CR10] Eitelberg DA, van Vliet J, Doelman JC, Stehfest E, Verburg PH (2016). Demand for biodiversity protection and carbon storage as drivers of global land change scenarios. Glob Environ Chang.

[CR11] Elliott J, Deryng D, Müller C, Frieler K, Konzmann M, Gerten D, Glotter M, Flörke M, Wada Y, Best N, Eisner S (2014). Constraints and potentials of future irrigation water availability on agricultural production under climate change. Proc Natl Acad Sci U S A.

[CR12] EUROSTAT (2013). Pocketbook on euro-Mediterranean statistics.

[CR13] EUROSTAT (2016) Agriculture statistics—North Africa and Eastern Mediterranean-statistics explained

[CR14] Evans RG, Sadler EJ (2008). Methods and technologies to improve efficiency of water use. Water Resour Res.

[CR15] Fader M, Shi S, von Bloh W, Bondeau A, Cramer W (2016). Mediterranean irrigation under climate change: more efficient irrigation needed to compensate increases in irrigation water requirements. Hydrol Earth Syst Sci.

[CR16] Fagúndez J, Olea PP, Tejedo P, Mateo-Tomás P, Gómez D (2016). Irrigation and maize cultivation erode plant diversity within crops in Mediterranean dry cereal agro-ecosystems. Environ Manag.

[CR17] Falkenmark M, Jägerskog A, Schneider K (2014). Overcoming the land–water disconnect in water-scarce regions: time for IWRM to go contemporary. Int J Water Resour D.

[CR18] FAO (2016) AQUASTAT website. FAO’s information system on water and agriculture. Food and Agriculture Organization of the United Nations

[CR19] Flörke M, Kynast E, Bärlund I, Eisner S, Wimmer F, Alcamo J (2013). Domestic and industrial water uses of the past 60 years as a mirror of socio-economic development: a global simulation study. Glob Environ Chang.

[CR20] Foley JA, Ramankutty N, Brauman KA, Cassidy ES, Gerber JS, Johnston M, Mueller ND, O’Connell C, Ray DK, West PC, Balzer C (2011). Solutions for a cultivated planet. Nature.

[CR21] Freier K, Finckh M, Schneider U (2014). Adaptation to new climate by an old strategy? Modeling sedentary and mobile pastoralism in semi-arid Morocco. Land.

[CR22] Fricko O, Havlik P, Rogelj J (2017). The marker quantification of the shared socioeconomic pathway 2: a middle-of-the-road scenario for the 21st century. Glob Environ Chang.

[CR23] Geerts S, Raes D (2009). Deficit irrigation as an on-farm strategy to maximize crop water productivity in dry areas. Agric Water Manag.

[CR24] Giannakopoulos C, Le Sager P, Bindi M, Moriondo M, Kostopoulou E, Goodess CM (2009). Climatic changes and associated impacts in the Mediterranean resulting from a 2 C global warming. Glob Planet Chang.

[CR25] Guiot J, Cramer W (2016). Climate change: the 2015 Paris agreement thresholds and Mediterranean basin ecosystems. Science.

[CR26] Hayashi A, Akimoto K, Tomoda T, Kii M (2013). Global evaluation of the effects of agriculture and water management adaptations on the water-stressed population. Mitig Adapt Strateg Glob Chang.

[CR27] Hijmans RJ, Cameron SE, Parra JL, Jones PG, Jarvis A (2005). Very high resolution interpolated climate surfaces for global land areas. Int J Climatol.

[CR28] Iglesias A, Mougou R, Moneo M, Quiroga S (2011). Towards adaptation of agriculture to climate change in the Mediterranean. Reg Environ Chang.

[CR29] IIASA (2016) SSP database (Shared Socioeconomic Pathways) - version 1.1

[CR30] IUCN (2015) World database on protected areas

[CR31] Jägermeyr J, Gerten D, Heinke J, Schaphoff S, Kummu M, Lucht W (2015). Water savings potentials of irrigation systems: global simulation of processes and linkages. Hydrol Earth Syst Sci.

[CR32] Jensen ME (2007). Beyond irrigation efficiency. Irrig Sci.

[CR33] Jiang L, O’Neill BC (2017). Global urbanization projections for the shared socioeconomic pathways. Glob Environ Chang.

[CR34] Joffre R, Rambal S, Ratte JP (1999). The dehesa system of southern Spain and Portugal as a natural ecosystem mimic. Agrofor Syst.

[CR35] Kc S, Lutz W (2017). The human core of the shared socioeconomic pathways: population scenarios by age, sex and level of education for all countries to 2100. Glob Environ Chang.

[CR36] Lankford B (2006). Localising irrigation efficiency. Irrig Drain.

[CR37] Latorre JG, García-Latorre J, Sanchez-Picón A (2001). Dealing with aridity: socio-economic structures and environmental changes in an arid Mediterranean region. Land Use Policy.

[CR38] Letourneau A, Verburg PH, Stehfest E (2012). A land-use systems approach to represent land-use dynamics at continental and global scales. Environ Model Softw.

[CR39] Licker R, Johnston M, Foley JA, Barford C, Kucharik CJ, Monfreda C, Ramankutty N (2010). Mind the gap: how do climate and agricultural management explain the ‘yield gap’ of croplands around the world?. Glob Ecol Biogeogr.

[CR40] Malek Ž, Verburg PH (2017). Mediterranean land systems: representing diversity and intensity of complex land systems in a dynamic region. Landsc Urban Plan.

[CR41] Médail F, Quézel P (1999). Biodiversity hotspots in the Mediterranean Basin: setting global conservation priorities. Conserv Biol.

[CR42] Mekonnen MM, Hoekstra AY (2012). A global assessment of the water footprint of farm animal products. Ecosystems.

[CR43] Moriondo M, Giannakopoulos C, Bindi M (2011). Climate change impact assessment: the role of climate extremes in crop yield simulation. Clim Chang.

[CR44] Mueller ND, Gerber JS, Johnston M, Ray DK, Ramankutty N, Foley JA (2012). Closing yield gaps through nutrient and water management. Nature.

[CR45] Neumann K, Verburg PH, Stehfest E, Müller C (2010). The yield gap of global grain production: a spatial analysis. Agric Syst.

[CR46] Olson DM, Dinerstein E, Wikramanayake ED (2001). Terrestrial ecoregions of the world: a new map of life on earth a new global map of terrestrial ecoregions provides an innovative tool for conserving biodiversity. Bioscience.

[CR47] Ortuño A, Hernández M, Civera S (2015). Golf course irrigation and self-sufficiency water in southern Spain. Land Use Policy.

[CR48] Qadir M, Sharma BR, Bruggeman A, Choukr-Allah R, Karajeh F (2007). Non-conventional water resources and opportunities for water augmentation to achieve food security in water scarce countries. Agric Water Manag.

[CR49] Riahi K, van Vuuren DP, Kriegler E (2017). The shared socioeconomic pathways and their energy, land use, and greenhouse gas emissions implications: an overview. Glob Environ Chang.

[CR50] Rost S, Gerten D, Hoff H, Lucht W, Falkenmark M, Rockström J (2009). Global potential to increase crop production through water management in rainfed agriculture. Environ Res Lett.

[CR51] Rudel TK, Schneider L, Uriarte M, Turner BL, DeFries R, Lawrence D, Geoghegan J, Hecht S, Ickowitz A, Lambin EF, Birkenholtz T (2009). Agricultural intensification and changes in cultivated areas, 1970–2005. Proc Natl Acad Sci U S A.

[CR52] Salmoral G, Willaarts BA, Garrido A, Guse B (2017). Fostering integrated land and water management approaches: evaluating the water footprint of a Mediterranean basin under different agricultural land use scenarios. Land Use Policy.

[CR53] Sauer T, Havlík P, Schneider UA, Schmid E, Kindermann G, Obersteiner M (2010). Agriculture and resource availability in a changing world: the role of irrigation. Water Resour Res.

[CR54] Schaich H, Kizos T, Schneider S, Plieninger T (2015). Land change in eastern Mediterranean wood-pasture landscapes: the case of deciduous oak woodlands in Lesvos (Greece). Environ Manag.

[CR55] Siebert S, Döll P, Hoogeveen J, Faures J-M, Frenken K, Feick S (2005). Development and validation of the global map of irrigation areas. Hydrol Earth Syst Sci.

[CR56] Siebert S, Henrich V, Frenken K, Burke J (2013). Update of the digital global map of irrigation areas to version 5.

[CR57] Smit B, Skinner MW (2002). Adaptation options in agriculture to climate change: a typology. Mitig Adapt Strateg Glob Chang.

[CR58] Souty F, Brunelle T, Dumas P (2012). The nexus land-use model version 1.0, an approach articulating biophysical potentials and economic dynamics to model competition for land-use. Geosci Model Dev.

[CR59] Sowers J, Vengosh A, Weinthal E (2010). Climate change, water resources, and the politics of adaptation in the Middle East and North Africa. Clim Chang.

[CR60] Taylor KE, Stouffer RJ, Meehl GA (2012). An overview of CMIP5 and the experiment design. Bull Am Meteorol Soc.

[CR61] Tieskens KF, Schulp CJE, Levers C, Lieskovský J, Kuemmerle T, Plieninger T, Verburg PH (2017). Characterizing European cultural landscapes: accounting for structure, management intensity and value of agricultural and forest landscapes. Land Use Policy.

[CR62] Trabucco A, Zomer RJ, Bossio DA, van Straaten O, Verchot LV (2008). Climate change mitigation through afforestation/reforestation: a global analysis of hydrologic impacts with four case studies. Agric Ecosyst Environ.

[CR63] UNEP/MAP-Plan Bleu (2010). State of the environment and development in the Mediterranean 2009.

[CR64] UNESCO (2006). *Water: a shared responsibility. World Water Development Report 2*.

[CR65] van Asselen S, Verburg PH (2013). Land cover change or land-use intensification: simulating land system change with a global-scale land change model. Glob Chang Biol.

[CR66] Vörösmarty CJ, McIntyre PB, Gessner MO (2010). Global threats to human water security and river biodiversity. Nature.

[CR67] Wisser D, Frolking S, Douglas EM, Fekete BM, Vörösmarty CJ, Schumann AH (2008). Global irrigation water demand: variability and uncertainties arising from agricultural and climate data sets. Geophys Res Lett.

[CR68] Wriedt G, Van der Velde M, Aloe A, Bouraoui F (2009). Estimating irrigation water requirements in Europe. J Hydrol.

[CR69] Wright B, Cafiero C (2011). Grain reserves and food security in the Middle East and North Africa. Food Sec.

[CR70] You L, Wood-Sichra U, Fritz S, Guo Z, See L, Koo L (2014) Spatial production allocation model (SPAM) 2005 v2.0. Available at mapspam. info. Accessed 12 Dec 2016

[CR71] Zomer RJ, Trabucco A, Bossio DA, Verchot LV (2008). Climate change mitigation: a spatial analysis of global land suitability for clean development mechanism afforestation and reforestation. Agric Ecosyst Environ.

[CR72] Zwart SJ, Bastiaanssen WGM (2004). Review of measured crop water productivity values for irrigated wheat, rice, cotton and maize. Agric Water Manag.

