# Supplementary material for: Adaptation of land management in the Mediterranean under scenarios of irrigation water use and availability
Source: Mitig Adapt Strateg Glob Chang. 2017 Sep 10;23(6):821–37. doi: 10.1007/s11027-017-9761-0 (PMC6054018; doi:10.1007/s11027-017-9761-0)
Supplement: Supplementary file 1 — (DOCX 4426 kb) [file 11027_2017_9761_MOESM1_ESM.docx]

**S1 -** **Mediterranean land systems map for 2010 (based on Malek & Verburg, 2017)**

**
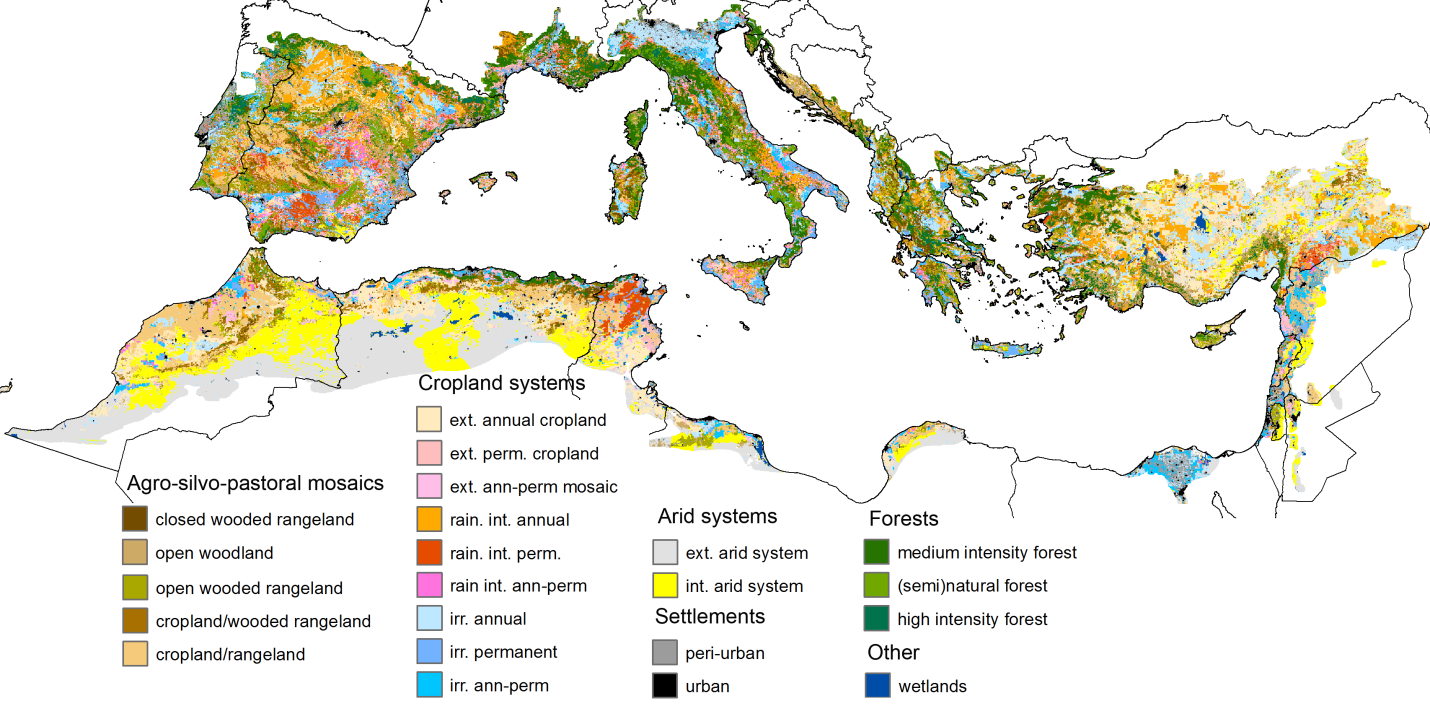
**

**S2 – Mediterranean land system characteristics tables per 4 km^2^ land system unit**

**Table S2.1 Mediterranean North – European Union**

| **Land System** | **Annual crops (t)** | **Permanent crops (t)** | **Livestock (nr)** | **Built up (ha)** | **Demand for water (m^3^)** |
| --- | --- | --- | --- | --- | --- |
| wetlands | 225.56 | 122.07 | 27.18 | 4.68 |  |
| medium inten. forest | 27.64 | 30.01 | 29.18 | 2.28 |  |
| (semi)natural forest | 21.11 | 22.21 | 31.12 | 1.00 |  |
| high inten. forest | 19.97 | 15.73 | 32.16 | 1.16 |  |
| ext. arid system | 21.08 | 32.54 | 28.75 | 5.35 |  |
| int. arid system | 26.98 | 67.03 | 57.90 | 3.04 |  |
| closed wooded rangel. | 35.87 | 25.82 | 83.27 | 0.96 |  |
| open woodland | 24.42 | 45.63 | 24.73 | 3.89 |  |
| open wooded rangel. | 36.59 | 42.38 | 66.23 | 1.70 |  |
| cropl./wooded rangel. | 77.10 | 49.78 | 67.12 | 2.64 |  |
| cropland/rangel. | 96.02 | 53.75 | 120.46 | 2.25 |  |
| exten. annual | 100.27 | 54.07 | 47.22 | 3.36 |  |
| exten. permanent | 15.60 | 303.27 | 36.13 | 5.04 |  |
| exten. mosaic | 70.76 | 140.29 | 48.72 | 3.60 |  |
| rainfed inten. annual | 1325.39 | 108.74 | 69.17 | 2.80 |  |
| rainfed inten. perm. | 345.27 | 1124.10 | 48.45 | 4.28 |  |
| rainfed inten. mosaic | 1076.36 | 413.48 | 54.47 | 3.44 |  |
| irrigated annual | 776.67 | 144.97 | 88.33 | 5.32 | 928.12 |
| irrigated permanent | 74.72 | 1180.42 | 38.98 | 6.24 | 853.28 |
| irrigated mosaic | 513.66 | 485.00 | 59.81 | 6.00 | 797.71 |
| peri-urban | 434.35 | 448.67 | 55.70 | 37.36 | 481.89 |
| urban | 334.02 | 330.53 | 29.92 | 112.12 | 358.25 |

Total crop production in 2010 (EUROSTAT, 2013; You *et al.*, 2014; EUROSTAT, 2016). Crop production category ”Irrigated” relates to irrigated and urban land systems, “Rainfed high” to rainfed intensive land systems, and “Rainfed low” to all remaining land systems.

|  | **Share (%)** | **Production (t)** |
| --- | --- | --- |
| **Annual crops** |  |  |
| Irrigated | 41.6 | 33104235 |
| Rainfed high | 48.7 | 35277415 |
| Rainfed low | 9.7 | 7036980 |
| Total annual |  | 75418630 |
| **Permanent crops** |  |  |
| Irrigated | 56.7 | 28885123 |
| Rainfed high | 27.2 | 13826362 |
| Rainfed low | 16.1 | 8197011 |
| Total permanent |  | 50908496 |

**Table S2.2 Mediterranean North – Western Balkans and Turkey**

| **Land System** | **Annual crops (t)** | **Permanent crops (t)** | **Livestock (nr)** | **Built up (ha)** | **Demand for water (m^3^)** |
| --- | --- | --- | --- | --- | --- |
| wetlands | 204.85 | 31.62 | 18.06 | 0.88 |  |
| medium inten. forest | 145.12 | 34.48 | 44.12 | 0.72 |  |
| (semi)natural forest | 173.65 | 1.09 | 30.31 | 1.28 |  |
| high inten. forest | 188.18 | 81.86 | 58.41 | 1.35 |  |
| ext. arid system | 238.04 | 23.84 | 21.04 | 0.30 |  |
| int. arid system | 272.86 | 32.29 | 51.86 | 0.68 |  |
| closed wooded rangel. | 197.85 | 48.71 | 134.11 | 0.44 |  |
| open woodland | 183.58 | 45.03 | 31.59 | 1.16 |  |
| open wooded rangel. | 224.65 | 88.03 | 79.88 | 1.18 |  |
| cropl./wooded rangel. | 359.68 | 62.94 | 69.59 | 1.16 |  |
| cropland/rangel. | 436.41 | 153.17 | 99.19 | 1.93 |  |
| exten. annual | 453.44 | 38.31 | 45.11 | 1.24 |  |
| exten. permanent | 101.26 | 1307.42 | 78.32 | 3.00 |  |
| exten. mosaic | 367.75 | 435.08 | 55.18 | 1.92 |  |
| rainfed inten. annual | 961.13 | 85.53 | 65.43 | 1.76 |  |
| rainfed inten. perm. | 117.16 | 2432.68 | 66.16 | 3.00 |  |
| rainfed inten. mosaic | 721.73 | 774.67 | 68.33 | 2.00 |  |
| irrigated annual | 719.41 | 92.06 | 81.23 | 2.52 | 1224.69 |
| irrigated permanent | 259.32 | 2445.74 | 103.03 | 5.80 | 1119.55 |
| irrigated mosaic | 566.59 | 805.33 | 93.83 | 3.36 | 1132.71 |
| peri-urban | 784.95 | 335.81 | 85.08 | 43.48 | 484.49 |
| urban | 565.23 | 289.54 | 72.56 | 134.80 | 308.18 |

Total crop production in 2010 (EUROSTAT, 2013; You *et al.*, 2014; EUROSTAT, 2016). Crop production category ”Irrigated” relates to irrigated and urban land systems, “Rainfed high” to rainfed intensive land systems, and “Rainfed low” to all remaining land systems.

|  | **Share (%)** | **Production (t)** |
| --- | --- | --- |
| **Annual crops** |  |  |
| Irrigated | 26.1 | 16645751 |
| Rainfed high | 27.9 | 17473003 |
| Rainfed low | 46.0 | 28688356 |
| Total annual |  | 62807110 |
| **Permanent crops** |  |  |
| Irrigated | 24.2 | 4232387 |
| Rainfed high | 38.9 | 6800665 |
| Rainfed low | 36.9 | 6456238 |
| Total permanent |  | 17489290 |

**Table S2.3 Mediterranean South – Middle East and North-East Africa**

| **Land System** | **Annual crops (t)** | **Permanent crops (t)** | **Livestock (nr)** | **Built up (ha)** | **Demand for water (m^3^)** |
| --- | --- | --- | --- | --- | --- |
| wetlands | 157.00 | 62.40 | 357.35 | 2.68 |  |
| medium inten. forest | 184.06 | 393.07 | 69.45 | 1.44 |  |
| (semi)natural forest | 16.87 | 125.74 | 69.34 | 0.00 |  |
| high inten. forest | 160.12 | 344.90 | 770.47 | 6.88 |  |
| ext. arid system | 15.87 | 18.92 | 5.65 | 0.90 |  |
| int. arid system | 49.64 | 57.54 | 61.58 | 2.12 |  |
| closed wooded rangel. | 150.32 | 371.09 | 215.29 | 4.00 |  |
| open woodland | 52.02 | 61.48 | 153.99 | 2.40 |  |
| open wooded rangel. | 58.87 | 74.51 | 95.09 | 2.68 |  |
| cropl./wooded rangel. | 311.41 | 256.66 | 116.10 | 4.40 |  |
| cropland/rangel. | 444.25 | 301.54 | 84.87 | 5.50 |  |
| exten. annual | 455.82 | 99.88 | 40.82 | 3.72 |  |
| exten. permanent | 272.29 | 833.01 | 76.54 | 1.68 |  |
| exten. mosaic | 393.46 | 415.37 | 67.61 | 4.64 |  |
| rainfed inten. annual | 2005.72 | 455.49 | 141.67 | 4.20 |  |
| rainfed inten. perm. | 925.66 | 2885.40 | 74.97 | 1.00 |  |
| rainfed inten. mosaic | 1609.23 | 1041.68 | 325.32 | 4.60 |  |
| irrigated annual | 2349.56 | 113.62 | 135.61 | 7.84 | 3510.68 |
| irrigated permanent | 931.55 | 1336.90 | 83.83 | 5.72 | 2952.11 |
| irrigated mosaic | 2152.74 | 499.65 | 196.17 | 7.96 | 3611.44 |
| peri-urban | 2040.21 | 341.79 | 191.33 | 38.36 | 2937.68 |
| urban | 1133.41 | 264.96 | 100.17 | 183.16 | 1169.11 |

Total crop production in 2010 (EUROSTAT, 2013; You *et al.*, 2014; EUROSTAT, 2016). Crop production category ”Irrigated” relates to irrigated and urban land systems, “Rainfed high” to rainfed intensive land systems, and “Rainfed low” to all remaining land systems.

|  | **Share (%)** | **Production (t)** |
| --- | --- | --- |
| **Annual crops** |  |  |
| Irrigated | 82.9 | 38857839 |
| Rainfed high | 4.4 | 1759748 |
| Rainfed low | 12.7 | 5027599 |
| Total annual |  | 45645186 |
| **Permanent crops** |  |  |
| Irrigated | 58.2 | 6560864 |
| Rainfed high | 8.0 | 900796 |
| Rainfed low | 33.8 | 3809004 |
| Total permanent |  | 11270664 |

**Table S2.4 Mediterranean South – North-West Africa**

| **Land System** | **Annual crops (t)** | **Permanent crops (t)** | **Livestock (nr)** | **Built up (ha)** | **Demand for water (m^3^)** |
| --- | --- | --- | --- | --- | --- |
| wetlands | 119.57 | 48.58 | 1154.39 | 0.48 |  |
| medium inten. forest | 56.44 | 56.90 | 64.33 | 1.32 |  |
| (semi)natural forest | 54.65 | 5.57 | 56.56 | 0.28 |  |
| high inten. forest | 86.68 | 57.82 | 75.31 | 1.91 |  |
| ext. arid system | 4.48 | 2.09 | 6.27 | 0.36 |  |
| int. arid system | 11.60 | 10.29 | 33.94 | 0.40 |  |
| closed wooded rangel. | 64.00 | 50.37 | 109.51 | 1.60 |  |
| open woodland | 38.39 | 33.69 | 37.20 | 2.26 |  |
| open wooded rangel. | 49.34 | 26.78 | 60.27 | 0.55 |  |
| cropl./wooded rangel. | 112.31 | 23.36 | 62.90 | 1.12 |  |
| cropland/rangel. | 130.50 | 64.19 | 73.88 | 1.25 |  |
| exten. annual | 125.33 | 17.15 | 29.51 | 1.32 |  |
| exten. permanent | 58.64 | 352.79 | 55.21 | 1.92 |  |
| exten. mosaic | 102.90 | 119.27 | 52.89 | 1.48 |  |
| rainfed inten. annual | 2344.57 | 49.25 | 51.72 | 2.88 |  |
| rainfed inten. perm. | 1186.20 | 450.19 | 89.80 | 0.92 |  |
| rainfed inten. mosaic | 1788.21 | 161.31 | 73.84 | 1.68 |  |
| irrigated annual | 1553.24 | 75.64 | 49.72 | 3.04 | 1354.95 |
| irrigated permanent | 822.48 | 1097.10 | 68.45 | 3.72 | 1757.67 |
| irrigated mosaic | 1106.78 | 459.71 | 59.33 | 3.76 | 1428.26 |
| peri-urban | 1097.65 | 267.02 | 90.25 | 46.16 | 424.76 |
| urban | 719.29 | 210.62 | 40.20 | 159.20 | 216.68 |

Total crop production in 2010 (EUROSTAT, 2013; You *et al.*, 2014; EUROSTAT, 2016). Crop production category ”Irrigated” relates to irrigated and urban land systems, “Rainfed high” to rainfed intensive land systems, and “Rainfed low” to all remaining land systems.

|  | **Share (%)** | **Production (t)** |
| --- | --- | --- |
| **Annual crops** |  |  |
| Irrigated | 52.8 | 17419629 |
| Rainfed high | 22.0 | 7242574 |
| Rainfed low | 25.2 | 8310512 |
| Total annual |  | 32972716 |
| **Permanent crops** |  |  |
| Irrigated | 49.0 | 5117591 |
| Rainfed high | 16.6 | 1733763 |
| Rainfed low | 34.4 | 3604275 |
| Total permanent |  | 10455630 |

**S3 – Explanatory variables used in calculating spatial preference maps using logistic regression**

| **Location Factor** | **Unit/description** | **Resolution** | **Date** | **Source** |
| --- | --- | --- | --- | --- |
| **Socio-economic** | | | | |
| Population density* | People/km^2^ | 1 km | 2010 | CIESIN (2015) |
| Rural population* | Rural population/km^2^ | 1 km | 2000 | CIESIN et al. (2011) |
| Market accessibility | Index (0-1) | 1 km | 2000-2010 | Verburg et al. (2011b) |
| Market influence | USD/person (ppp) | 1 km | 2000-2010 | Verburg et al. (2011b) |
| Accessibility | Distance to roads (m) | vector | 1999 | NGIA (2015) |
| **Soil** | | | | |
| Drainage | Drainage class | 1 km | 2010 | Hengl et al. (2014) |
| Sand content | Sand mass in % | 1 km | 2010 | Stoorvogel et al. (2016) |
| Clay content | Clay mass in % | 1 km | 2013 | Stoorvogel et al. (2016) |
| Cation Exchange Capacity (CEC) | cmol/kg | 1 km | 2010 | Hengl et al. (2014) |
| pH | log(h+) | 1 km | 2010 | Hengl et al. (2014) |
| Organic carbon content | g/kg in the top 50 cm | 1 km | 2013 | Stoorvogel et al. (2016) |
| Soil depth | cm | 1 km | 2013 | Stoorvogel et al. (2016) |
| **Terrain** | | | | |
| Altitude | m above sea level | 1 km | 2005 | Hijmans et al. (2005) |
| Slope | Slope degrees | 1 km | 2005 | derived from Hijmans et al. (2005) |
| **Climate** | | | | |
| Precipitation* | annual precipitation (sum of monthly means) in mm | 1 km | 2005 | Hijmans et al. (2005) |
| Temperature* | Temperature (mean of monthly means) Celsius degree | 1 km | 2005 | Hijmans et al. (2005) |
| Solar radiation | Horizontal surface irradiation (kWh/m^2^), 1998-2011 mean | 1.5 arc minute | 2012 | Huld et al. (2012) |
| **Other** |  |  |  |  |
| Potential Evapotranspiration (PET)* | annual PET in mm | 1 km | 2007 | Zomer et al. (2008) |
| Potential vegetation | Pot. vegetation classes | 10 km | 2000 | Ellis & Ramankutty (2008) |

* dynamic factor, updated annually

**S4 – example of spatial preference maps**


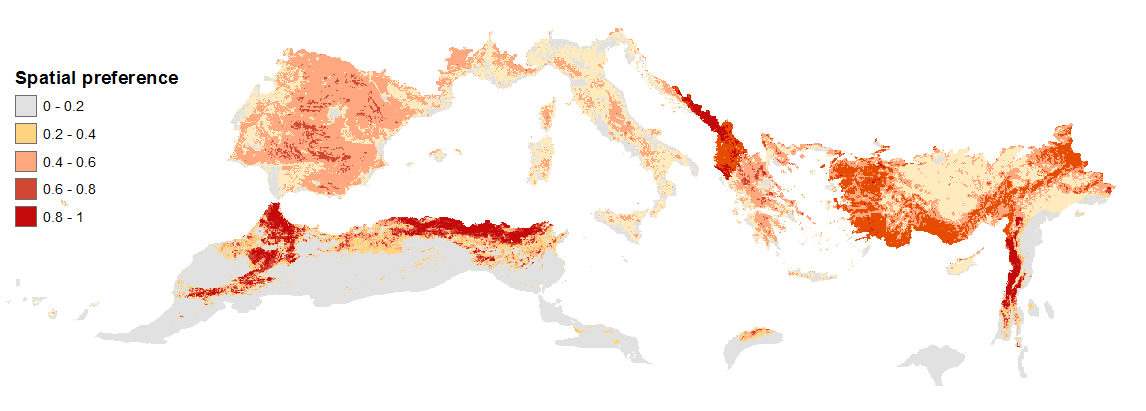


S4.1 Spatial preference map for the cropland/wooded rangeland mosaic system


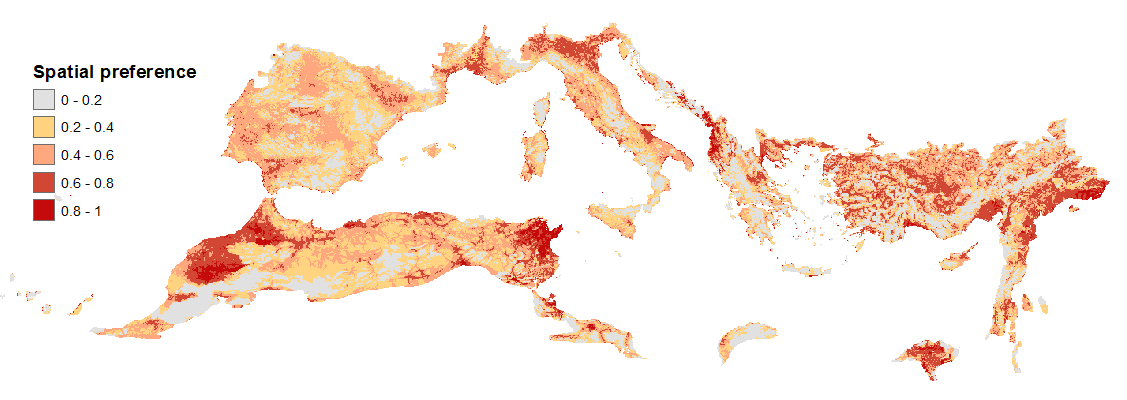


S4.2 Spatial preference map for the irrigated annual cropland system

**S5 – Changes to urban population**

**
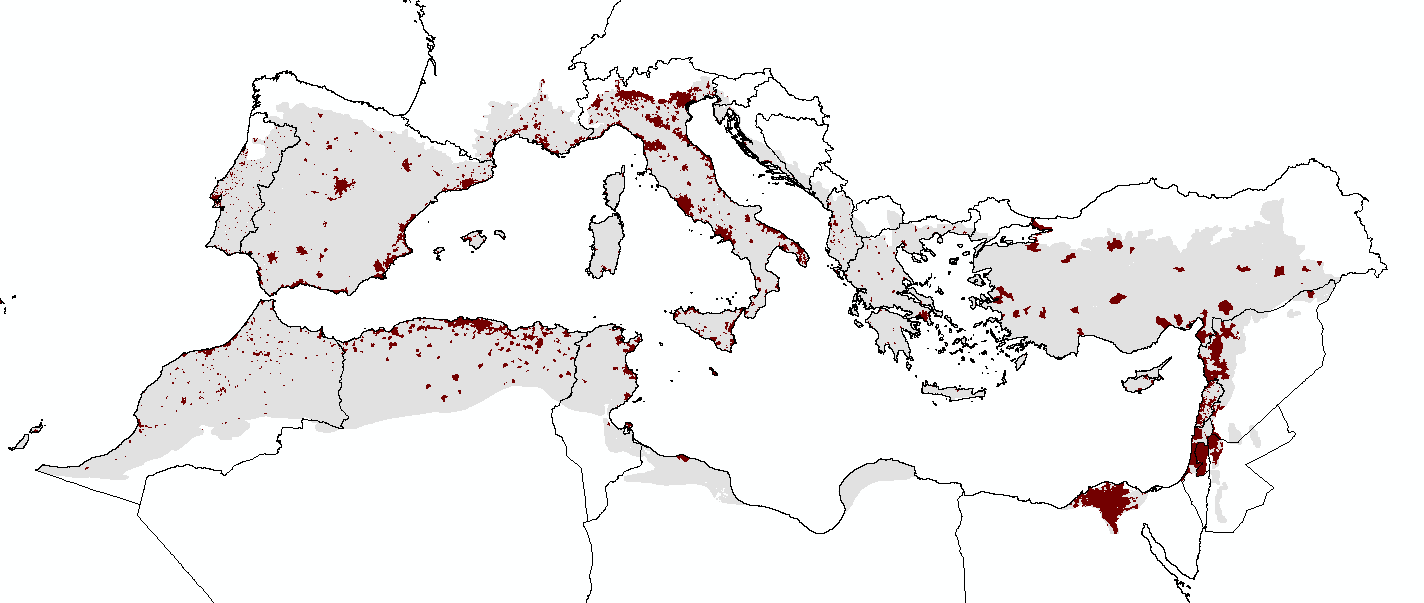
**

S5 Areas with high population density where urban population change trends based on the SSP2 scenario were applied (Kc & Lutz, 2017; Jiang & O’Neill, 2017)

**S6 – CMIP5 simulations of the RCP4.5 scenario used to update precipitation, temperature, PET and AI maps**

| **Model** | **Institution** |
| --- | --- |
| ACCESS1-0 | Commonwealth Scientific and Industrial Research Organisation (CSIRO) and Bureau of Meteorology, Australia |
| bcc-csm1-1 | Beijing Climate Center, China Meteorological Administration |
| CCSM4 | National Center for Atmospheric Research, USA |
| CESM1(CAM5.1, FV2) | National Science Foundation, Department of Energy, National Center for Atmospheric Research, USA |
| CNRM-CM5 | Centre National de Recherches Meteorologiques / Centre Europeen de Recherche et Formation Avancees en Calcul Scientifique, France |
| GFDL-CM3 | NOAA, Geophysical Fluid Dynamics Laboratory, USA |
| GFDL-ESM2G | NOAA, Geophysical Fluid Dynamics Laboratory, USA |
| GISS-E2-R | NASA Goddard Institute for Space Studies, USA |
| HadGEM2-AO | National Institute of Meteorological Research / Korea Meteorological Administration |
| HadGEM2-CC | Met Office Hadley Centre, UK |
| HadGEM2-ES | Met Office Hadley Centre / Instituto Nacional de Pesquisas Espaciais) |
| INM-CM4 | Institute for Numerical Mathematics, Russia |
| IPSL-CM5A-LR | Institut Pierre-Simon Laplace, France |
| MIROC-ESM-CHEM | Japan Agency for Marine-Earth Science and Technology, Atmosphere and Ocean Research Institute (The University of Tokyo), and National Institute for Environmental Studies |
| MIROC-ESM | Japan Agency for Marine-Earth Science and Technology, Atmosphere and Ocean Research Institute (The University of Tokyo), and National Institute for Environmental Studies |
| MIROC5 | Atmosphere and Ocean Research Institute (The University of Tokyo), National Institute for Environmental Studies, and Japan Agency for Marine-Earth Science and Technology |
| MPI-ESM-LR | Max Planck Institute for Meteorology (MPI-M), Germany |
| MRI-CGCM3 | Meteorological Research Institute, Japan |
| NorESM1-M | Norwegian Climate Centre |


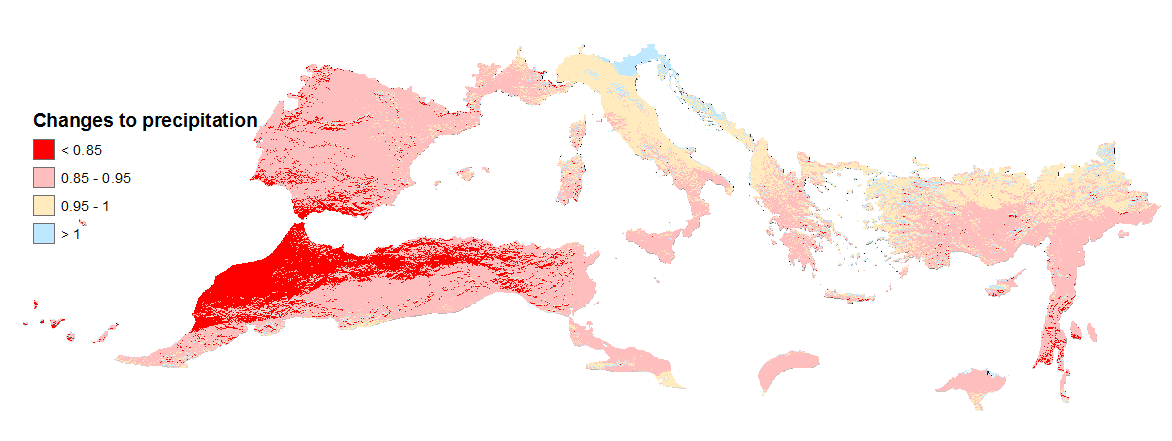


S6.1 2010-2050 changes in precipitation based on the mean of 19 CMIP5 simulations of the RCP4.5 for the Mediterranean ecoregion


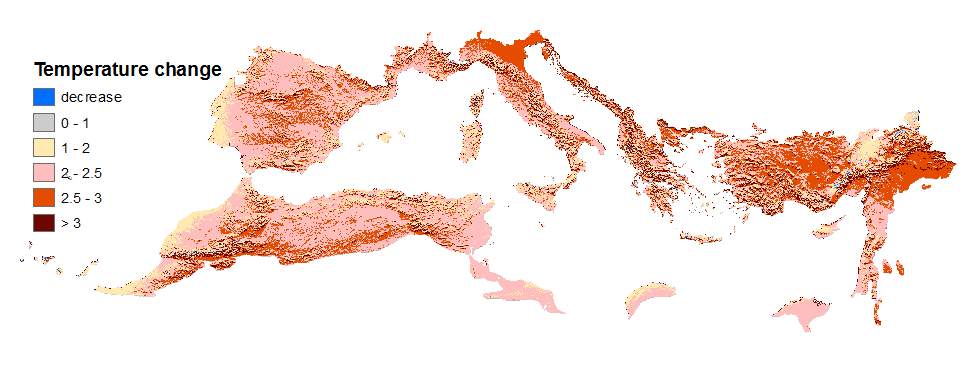


S6.2 2010-2050 changes in temperature in °C based on the mean of 19 CMIP5 simulations of the RCP4.5 for the Mediterranean ecoregion

**S7 – PET and AI calculation and map example**

Potential evapotranspiration (PET) represents the ability of the atmosphere to remove water through evapotranspiration processes, and was introduced by the FAO (Allen & FAO, 1998; Trabucco *et al.*, 2008). We used the Hargreaves model to calculate PET in this study (Hargreaves & Allen, 2003):

$$PET=0.0023\times RA\times(Tmean+17.8)\times{TD}^{0.5}$$

PET – monthly average PET (mm/year)

RA – annual extra-terrestrial radiation, radiation on top of atmosphere expressed (mm/year)

Tmean – annual mean temperature (°C)

TD – annual mean daily temperature range (°C)

We used spatial distributions on RA, Tmean and TD from the Worldclim dataset (Hijmans *et al.*, 2005). Future temperature spatial distributions were acquired by calculating the mean from 19 CMIP5 simulations of the RCP4.5 scenario (S7).


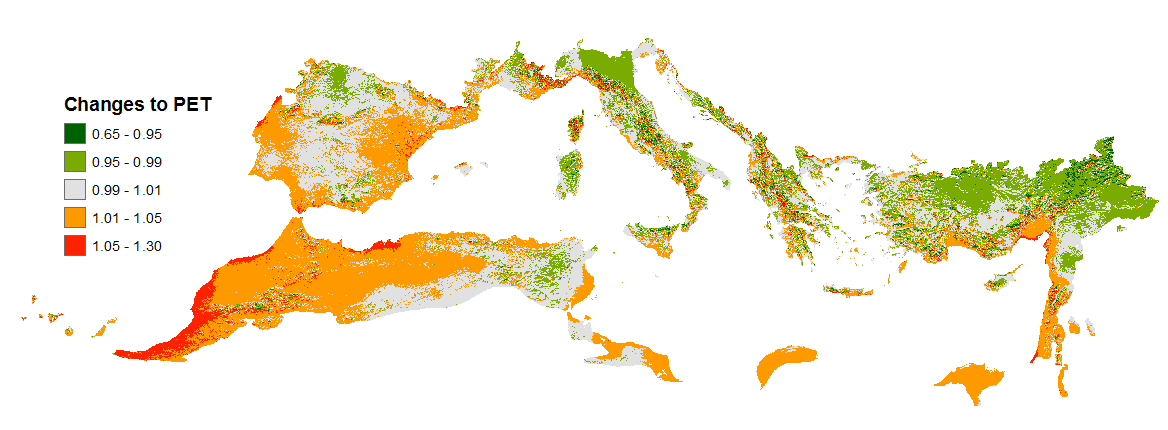


S7.1 2010-2050 changes in annual PET based on the 19 CMIP5 simulations of the RCP4.5 for the Mediterranean ecoregion. The future decrease in the temperature range is balancing the increase in the mean temperature, leading to a decrease in PET in some areas (marked as green on the figure).

The Aridity index serves as an indicator to quantify precipitation deficits over atmospheric water demand (UNEP, 1997; Zomer *et al.*, 2008). It is defined as a function of precipitation and potential evapotranspiration (PET):

$$AI= \frac{MAP}{MAE}$$

AI – aridity index

MAP – mean annual precipitation (mm/year)

MAE – mean annual potential evapotranspiration (PET, mm/year)


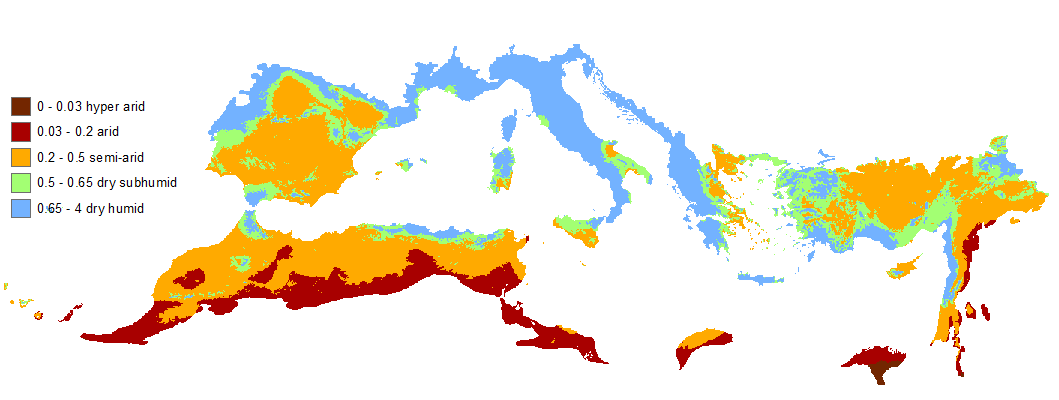


S7.2 Aridity index in 2010


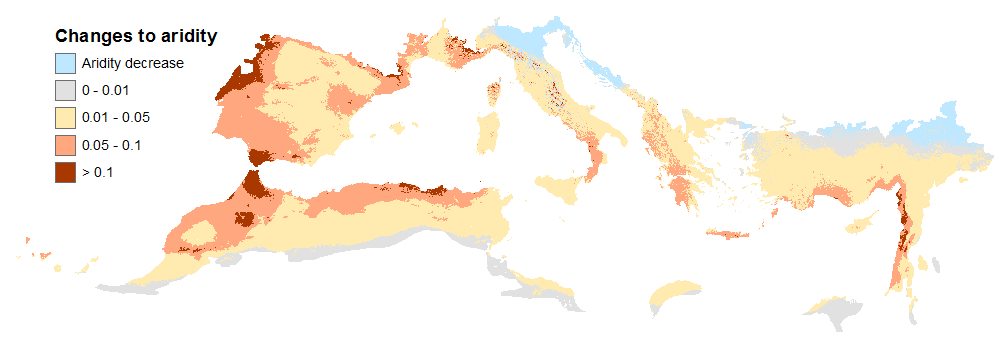


S7.3 Changes to aridity in 2050 based on the 19 CMIP5 simulations of the RCP4.5 for the Mediterranean ecoregion

**S8 – technical details of the CLUMondo application**

Table continues on next page

| **Model setting** | **Description** |
| --- | --- |
| **Dynamic explanatory variables** | Change every year |
| Population density | Growth rates based on SSP2 projections of Jiang and O’Neill (2017) applied to high population areas only (> 250 per km^2^, S4) |
| Rural population density | Change rates (growth/decrease) based on SSP2 projections of Jiang and O’Neill (2017) applied to rural population density map |
| Temperature, climate, potential evapotranspiration | Based on RCP4.5 climate change maps (mean of all model runs for RCP4.5). PET calculated using PET equations (S5, S6) |
| Aridity index | Calculated using the AI equation, dynamic for every year (RCP4.5) (S6) |
| **Demands** |  |
| Annual crops | Annual crops follow the SSP2 marker scenario for food production. |
| Permanent crops | Permanent crops follow the SSP2 marker scenario for food production. |
| Livestock | Livestock numbers follow the SSP2 scenario for livestock numbers. |
| Built up areas | Demand linked to population change (SSP2). |
| **Land system specific settings** |  |
| *Wetlands* |  |
| Supply | Same supply as baseline. |
| Spatial pattern/change process | Protected – no change possible |
| *Settlement systems* |  |
| Supply | 1. Remains the same as in the baseline for built up areas 2. Efficiency improvement for crop production (closing yield gaps in EU, 90 % of potential yield achieved in other regions) 3. Increase in livestock output by 5 % due to improvements in breeds and herd fattening. |
| Spatial pattern/change process | 1. Neighborhood of land system allocation consists of 2 neighboring cells. 2. Conversion to urban only possible in non-protected areas. 3. Urban land has absolute priority. Any system (except natural forests and wetlands) can be converted to a settlement. |
| *Forest systems* |  |
| Supply | N.A. |
| Spatial pattern/change process | 1. All forest systems (including closed wooded rangeland) can change to open woodland in areas with an aridity index < 0.65. 2. Forest expansion (open woodlands to forests) only possible in areas with an AI > 0.65. |
| *Rainfed intensive* |  |
| Supply | 1. Yields reach 75 % of potential yield - higher yields in the region often not possible without irrigation (Mueller *et al.*, 2012) 2. Increase in livestock output by 5 % due to improvements in breeds and herd fattening. |
| Spatial pattern/change process | 1. Possible only in areas with AI > 0.2. In areas with 0.2<AI<0.65, there is a decrease in probability of rainfed intensive areas (-0.05) to account for extreme climate events. 2. Not possible in protected areas. 3. Can be transformed to less intensive systems after 10 years (annual crops) or 15 years (permanent crops) |
| *Extensive cropland* |  |
| Supply | 1. 50 % of potential yield achieved due to crop change or labor intensification (due to rural development policies and small farm promotion). 2. Increase in livestock output by 5 % due to improvements in breeds and herd fattening. |
| Spatial pattern/change process | 1. No spatial limitations. 2. Conversions to woodlands after being inactive for 10 years (abandonment). |
| *Irrigated cropland* |  |
| Supply | 1. Yield gaps closed in EU, in other regions yields reach 90 % of potential yield. 2. Output of annual crops decreases by 4% in the deficit irrigation scenario 3. Improved irrigation efficiency depending on the scenario. 4. Increase in livestock output by 5 % due to improvements in breed and herd fattening |
| Spatial pattern/change process | Irrigated areas limited by amount of available water resources (by region), and by water level (current water withdrawal and 25% reduction in withdrawal). |
| Mosaic systems |  |
| Supply | Increase in livestock output by 5 % due to improvements in breeds and herd fattening |
| Spatial pattern/change process | Mosaic systems can occur everywhere, based on the spatial preference map (also in protected areas) |
| Arid systems |  |
|  | 1. Grazing intensification in arid systems allowed. 2. Increase in livestock output by 5 % due to improvements in breeds and herd fattening. |
| Other |  |
| Biodiversity/nature protection | Intensification, cropland expansion, irrigation and urban expansion is not possible in national parks and similar protected areas. |

**S9 – 2050 land system distribution under different irrigation efficiency scenarios**


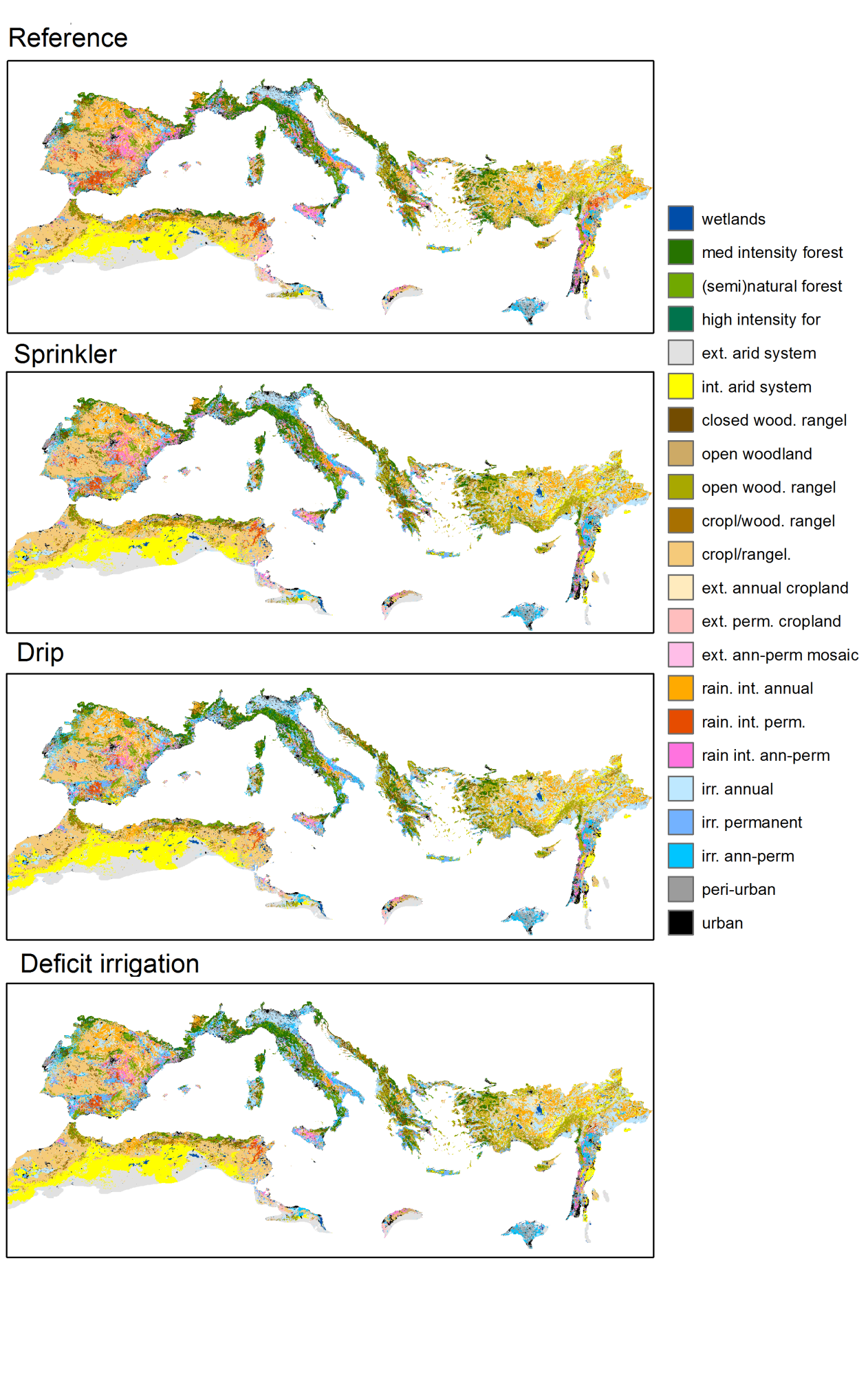


S9.1 Future land system scenarios under current water withdrawal

**
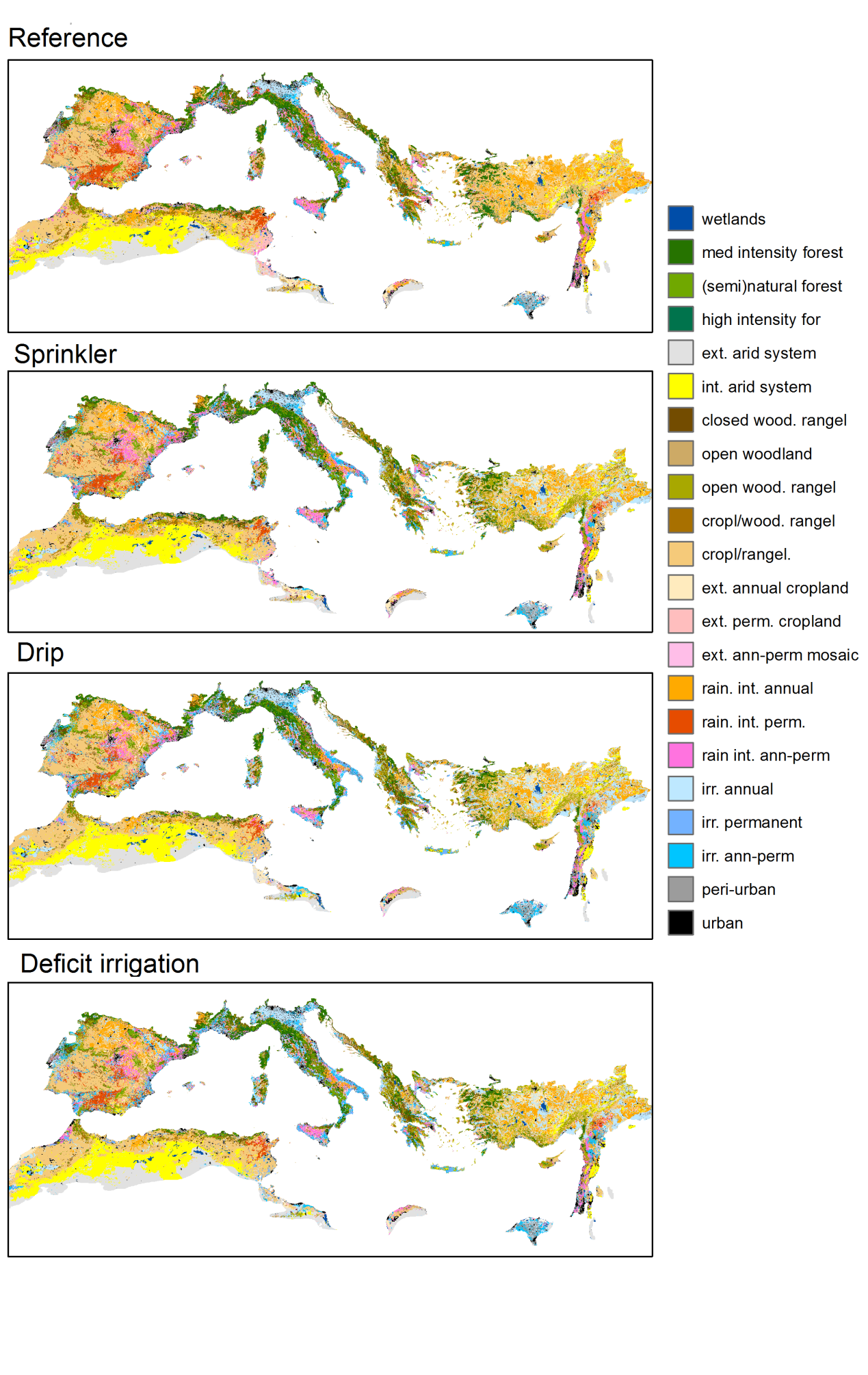
**

S9.2 Future land system scenarios under reduced water withdrawal

**S10 – Allocated adaptation options in land management under different scenarios of irrigation efficiency and water withdrawal in 1000 km^2^**

|  | **Reference** | | **Sprinkler** | | **Drip** | | **Deficit** | |
| --- | --- | --- | --- | --- | --- | --- | --- | --- |
| Process | current | reduced | current | reduced | current | reduced | current | reduced |
| Implementing irrigation | 15.4 | 7.7 | 78.2 | 39.0 | 136.5 | 85.5 | 138.9 | 84.9 |
| Cropland intensification | 92.8 | 131.0 | 70.5 | 97.7 | 64.0 | 75.6 | 60.0 | 76.2 |
| Cropland expansion | 41.5 | 44.2 | 26.4 | 38.3 | 17.4 | 30.5 | 22.5 | 34.0 |
| Cropland abandonment | 64.2 | 28.8 | 82.6 | 65.7 | 67.0 | 48.0 | 75.9 | 52.3 |
| Decrease in intensity | 78.8 | 181.3 | 50.1 | 103.6 | 40.4 | 79.6 | 39.8 | 81.9 |

**S11 – Pressure on freshwater resources under different irrigation efficiency scenarios**

We compared all scenarios in terms of the pressure on freshwater resources (PFR). PFR is used as a metric to describe water stress and the state of freshwater. It is defined as the ratio between total irrigation water withdrawal and available freshwater resources (FAO, 2016). A PFR higher than 25% prevents normal biological functioning of water leading to water stress as a potentially development limiting factor (Arnell, 1999; FAO, 2016).

Table S11. Pressure on freshwater resources under different irrigation efficiency scenarios in %

|  | **Reference** | | **Sprinkler** | | **Drip** | | **Deficit** | |
| --- | --- | --- | --- | --- | --- | --- | --- | --- |
| Subregion | current | reduced | current | reduced | current | reduced | current | reduced |
| Western Balkans and Turkey | 11.8 | 2.9 | 11.8 | 8.8 | 11.2 | 8.9 | 11.8 | 8.8 |
| European Union | 8.7 | 6.2 | 10.1 | 7.6 | 10.2 | 7.6 | 10.1 | 7.6 |
| Middle East and NE Africa | 93.9 | 70.9 | 92.3 | 71.2 | 77.0 | 71.4 | 80.0 | 71.4 |
| NW Africa | 21.1 | 11.1 | 26.9 | 22.7 | 23.2 | 20.7 | 22.2 | 21.0 |

**References**

Allen RG, FAO (eds.) (1998) Crop evapotranspiration: guidelines for computing crop water requirements. Food and Agriculture Organization of the United Nations, Rome, 300 pp.

Arnell N (1999) Climate change and global water resources. Global Environmental Change, 9, S31–S49.

CIESIN (2015) Gridded Population of the World, Version 4. Center for International Earth Science Information Network - CIESIN - Columbia University. NASA Socioeconomic Data and Applications Center (SEDAC).

CIESIN, IFPRI, CIAT (2011) Global Rural-Urban Mapping Project, Version 1: Population Density Grid. Center for International Earth Science Information Network - CIESIN - Columbia University, International Food Policy Research Institute - IFPRI, The World Bank, and Centro Internacional de Agricultura Tropical - CIAT.

Ellis EC, Ramankutty N (2008) Putting people in the map: anthropogenic biomes of the world. *Frontiers in Ecology and the Environment*, **6**, 439–447.

EUROSTAT (2013) *Pocketbook on Euro-Mediterranean statistics*. Publications office of the European Union, Luxembourg.

EUROSTAT (2016) Agriculture statistics - North Africa and Eastern Mediterranean - Statistics Explained.

FAO (2016) AQUASTAT website. FAO’s Information System on Water and Agriculture. Food and Agriculture Organization of the United Nations.

Hargreaves G, Allen R (2003) History and Evaluation of Hargreaves Evapotranspiration Equation. *Journal of Irrigation and Drainage Engineering*, **129**, 53–63.

Hengl T, de Jesus JM, MacMillan RA et al. (2014) SoilGrids1km — Global Soil Information Based on Automated Mapping. *PLoS ONE*, **9**, e105992.

Hijmans RJ, Cameron SE, Parra JL, Jones PG, Jarvis A (2005) Very high resolution interpolated climate surfaces for global land areas. *International Journal of Climatology*, **25**, 1965–1978.

Huld T, Müller R, Gambardella A (2012) A new solar radiation database for estimating PV performance in Europe and Africa. *Solar Energy*, **86**, 1803–1815.

Jiang L, O’Neill BC (2017) Global urbanization projections for the Shared Socioeconomic Pathways. Glob Environ Chang, **42**, 193-199

Kc S, Lutz W (2017) The human core of the shared socioeconomic pathways: Population scenarios by age, sex and level of education for all countries to 2100. Glob Environ Chang, **42**, 181-192

Mueller ND, Gerber JS, Johnston M, Ray DK, Ramankutty N, Foley JA (2012) Closing yield gaps through nutrient and water management. *Nature*, **490**, 254–257.

NGIA (2015) VMap0 data. National Geospatial Intelligence Agency. http://gis-lab.info/qa/vmap0-eng.html.

Pouzols FM, Toivonen T, Di Minin E et al. (2014) Global protected area expansion is compromised by projected land-use and parochialism. *Nature*, **516**, 383–386.

Stoorvogel JJ, Bakkenes M, Temme AJAM, Batjes NH, ten Brink B (2016) S-World: a Global Soil Map for Environmental Modelling: S-World: a Global Soil Map for Environmental Modelling. *Land Degradation & Development*, **28**, 22-33.

Trabucco A, Zomer RJ, Bossio DA, van Straaten O, Verchot LV (2008) Climate change mitigation through afforestation/reforestation: A global analysis of hydrologic impacts with four case studies. *Agriculture, Ecosystems & Environment*, **126**, 81–97.

UNEP (1997) *World atlas of desertification*. United Nations Environment Programme, London ; New York : New York.

Verburg PH, Ellis EC, Letourneau A (2011) A global assessment of market accessibility and market influence for global environmental change studies. *Environmental Research Letters*, **6**, 034019.

You L, Wood-Sichra U, Fritz S, Guo Z, See L, Koo L (2014) Spatial Production Allocation Model (SPAM) 2005 v2.0. *Available at mapspam. info. Accessed Dec, 12,* 2016

Zomer RJ, Trabucco A, Bossio DA, Verchot LV (2008) Climate change mitigation: A spatial analysis of global land suitability for clean development mechanism afforestation and reforestation. *Agriculture, Ecosystems & Environment*, **126**, 67–80.
